# A Novel DCL2-Dependent Micro-Like RNA *Vm*-PC-3p-92107_6 Affects Pathogenicity by Regulating the Expression of *Vm*-*VPS10* in *Valsa mali*

**DOI:** 10.3389/fmicb.2021.721399

**Published:** 2021-10-01

**Authors:** Feiran Guo, Jiahao Liang, Ming Xu, Gao Zhang, Lili Huang, Hao Feng

**Affiliations:** State Key Laboratory of Crop Stress Biology for Arid Areas and College of Plant Protection, Northwest A&F University, Yangling, China

**Keywords:** Dicer, micro-like RNA, pathogenicity, RNA silencing, *Valsa mali*

## Abstract

Dicer proteins are mainly responsible for generating small RNAs (sRNAs), which are involved in gene silencing in most eukaryotes. In previous research, two DCL proteins in *Valsa mali*, the pathogenic fungus causing apple tree *Valsa* canker, were found associated with both the pathogenicity and generation of sRNAs. In this study, the differential expression of small interfering RNAs (siRNAs) and miRNA-like RNAs (milRNAs) was analyzed based on the deep sequencing of the wild type and *Vm*-*DCL2* mutant, respectively. Overall, the generation of 40 siRNAs and 18 milRNAs was evidently associated with *Vm*-*DCL2.* The target genes of milRNAs were then identified using degradome sequencing; according to the prediction results, most candidate targets are related to pathogenicity. Further, expression of *Vm*-PC-3p-92107_6 was confirmed in the wild type but not in the *Vm*-*DCL2* mutant. Moreover, the pathogenicity of *Vm*-PC-3p-92107_6 deletion mutants (Δ*Vm*-PC-3p-92107_6) and the over-expression transformants (*Vm*-PC-3p-92107_6-OE) was significantly increased and decreased, respectively. Based on those degradome results, vacuolar protein sorting 10 (*Vm*-*VPS10*) was identified as the target of *Vm*-PC-3p-92107_6. Co-expression analysis in tobacco leaves further confirmed that *Vm*-PC-3p-92107_6 could suppress the expression of *Vm*-*VPS10*. Meanwhile, the expression levels of *Vm*-PC-3p-92107_6 and *Vm*-*VPS10* displayed divergent trends in Δ*Vm*-PC-3p-92107_6 and *Vm*-PC-3p-92107_6-OE, respectively. Perhaps most importantly, Δ*Vm*-*VPS10* featured a significant reduction in pathogenicity. Taken together, our results indicate that a DCL2-dependent milRNA *Vm*-PC-3p-92107_6 plays roles in pathogenicity by regulating the expression of *Vm*-*VPS10*. This study lays a foundation for the comprehensive analysis of pathogenic mechanisms of *V. mali* and deepens our understanding of the generation and function of fungal sRNA.

## Introduction

RNA interference (RNAi) is a conserved gene silencing mechanism in most eukaryotes (Dahlmann and Kueck, 2015). In the RNAi pathway, Dicer or Dicer-like (DCL) proteins are responsible for cutting long double-stranded RNAs, or those RNAs with a typical stem-loop structure, into small RNAs (sRNAs; Shabalina and Koonin, 2008). These sRNAs are then loaded into AGO proteins, after which the guide strand sRNA directs the RNA-induced silencing complex (RISC) to match and cleave the complementary mRNAs or suppress their translation (Fire et al., 1998; Chang et al., 2012). There are two major regulatory classes of sRNAs: short interfering RNAs (siRNAs) and microRNAs (miRNAs; Gent et al., 2010). The siRNAs generally guide gene silencing by binding perfectly to the complementary mRNAs. In addition to degrading the target gene’s mRNA, miRNAs can also suppress the translation of that target gene, but miRNAs do not need to be perfect complementarity to the mRNA (Nakayashiki et al., 2006). Both siRNAs and miRNAs play important roles in most eukaryotes with respect to the growth, development, and response to biotic or abiotic stresses (Doench et al., 2003; Ghildiyal and Zamore, 2009).

Fungi are fundamental evolved branch of eukaryotic organisms. Given that core RNAi components have been found in a wide range of fungal species, corresponding functional RNAi pathways may also exist in fungi (Mochizuki and Gorovsky, 2005). Previous studies have shown that Dicer proteins might be involved in various biological processes in fungi. The association of Dicer proteins with siRNA production and vegetative growth of mycelia were confirmed in both *Neurospora crassa* and *Mucor circinelloides* (Catalanotto et al., 2004; Nicolas et al., 2007, 2010). Yet the functioning of DCL proteins was redundant in *N. crassa* (Catalanotto et al., 2004) while their functions in *M. circinelloides* were divergent. For example, DCL1 is mainly responsible for regulating vegetative development and other biological functions of mycelia, while DCL2 is mainly responsible for generating sRNAs (Nicolas et al., 2007; de Haro et al., 2009).

The generation of sRNAs is essential because they act as the source switch of RNAi. It is now widely accepted that generation mechanisms of sRNAs are very complex in fungi (Lee et al., 2010; Jin et al., 2019). In *N. crassa*, the siRNAs could be generated in both Dicer-dependent and Dicer-independent pathways (Lee et al., 2010). Further, the production of milR-1 was completely dependent on Dicer, QDE-2, QIP, and MRPL3, while that of milR-2 did not depend on Dicers but did require QDE-2; conversely, the production of milR-3 was completely dependent on Dicers but the biogenesis of milR-4 depended only partly on Dicers (Lee et al., 2010). Moreover, in *Verticillium dahlia*, the biogenesis of *Vd*milR1 requires an RNase III domain-containing protein VdR3, but not Dicer-like or Argonaute proteins (Jin et al., 2019).

Since the discovery of sRNAs in fungi, some of their various functions have been revealed. In *N. crassa*, sRNAs play key roles in genome defense and gene regulation *via* post-transcriptional gene silencing activity (Fulci and Macino, 2007). Nonetheless, sRNAs could also affect the vegetative growth, pathogenicity, and toxin synthesis of certain fungi. For example, the milRNAs in *Penicillium marneffei* are capable of regulating the growth and development of mycelia, in addition to their participation in fungal pathogenicity and hormone secretion (Lau et al., 2013). Another role of milRNAs is to regulate mycotoxin biosynthesis and mycelium growth, as demonstrated in *Aspergillus flavus* (Bai et al., 2015), while a study of *Curvularia lunata* revealed that its milRNAs might contribute to pathogen infection and mycelial growth (Liu et al., 2016). In *Fusarium oxysporum* f. sp. *niveum*, the *Fon*-miR7696a-3p and *Fon*-miR6108a were found associated with trichothecene and NEP1 biosynthesis (Jiang et al., 2017). Recently, the milR236 of *Magnaporthe oryzae* was found able to influence both appressorium formation and pathogenicity by regulating the expression of the histone acetyltransferase gene *MoHat1* (Li et al., 2020). Other works have shown that *Pst*milR1 of *Puccinia striiformis* sp. *tritici* could inhibit the plant immune response by suppressing the expression of PR2 in a cross-kingdom RNAi pathway (Wang et al., 2017). More importantly, several DCL2-dependent milRNAs in *Fusarium graminearum* are known to be relevant to sexual reproduction (Zeng et al., 2018). In the fungus *V. dahliae*, a Dicer-independent milRNA, *Vd*milR1, was shown to regulate its pathogenicity by promoting the histone H3K9 methylation of *VdHy1* and transcriptional inhibition of the 3′ UTR of the protein-coding gene *VdHy1* (Jin et al., 2019). Nevertheless, the generation and functional mechanism of sRNAs in fungi are still largely unknown.

*Valsa mali* (*Cytospora* spp.) is a critical species of ascomycete as it causes the severest of apple tree trunk disease (Wang et al., 2014). Previous studies revealed that key RNAi components DCL and AGO proteins are involved in the stress responses and pathogenicity of this fungal species (Feng et al., 2017a,b). In particular, the *Vm*-*DCL2* deletion mutants showed a significant reduction in pathogenicity and sRNA abundance (Feng et al., 2017a). In the present study, the sRNAs in both the wild type and *Vm*-*DCL2* deletion mutant were analyzed to identify the *Vm*-*DCL2*-dependent sRNAs. We prove that a *Vm*-*DCL2*-dependent milRNA, *Vm*-PC-3p-92107_6, is involved in pathogenicity by regulating the expression of the vesicle pathway-related gene *Vm*-*VPS10*.

## Materials and Methods

### Strains and Growth Conditions

The *V. mali* wild-type strain 03–8 and *Vm*-*DCL2* deletion mutant strains were kept in storage by the Research Team of Pathogen Biology and Integrated Control of Fruit Trees, at the College of Plant Protection, Northwest A&F University, China. All strains were cultured in a PDA medium at 25°C in darkness. *Escherichia coli* DH5α was cultured in LB medium at 37°C.

### sRNAs Sequence Data Analysis

The sRNAs libraries of wild-type 03–8 (MVm) and *Vm*-*DCL2* deletion mutant (MD2) strains were constructed in previous study (Feng et al., 2017a). Raw data were first processed through custom Perl and python scripts. During this step, clean data were obtained by removing reads containing ploy-N, reads with 5′ adapter contaminants, reads without 3′ adapters or insert tags, reads containing ploy A, T, G, or C, and low-quality reads from the raw data. The clean reads were mapped into the reference sequence using Bowtie (Langmead et al., 2009) without a mismatch to confirm the sequence accuracy. The number of total unique sRNAs from the different samples was calculated to compare the difference between the MVm and MD2. To preliminarily estimate the sRNAs varieties, the length distribution of sRNAs in MVm and MD2 was also analyzed.

### Expression Profiles of *Vm*-milRNAs

Raw data were processed using the Illumina pipeline filter (Solexa 0.3). The ensuing data were subjected to the ACGT101-miR (LC Sciences, Houston, TX, United States) to remove any adapter dimers, junk, common RNA families, low complexity, and repeats. The specific screening process applied to milRNA in *V. mali* was consistent with that already described in a previous study (Xu et al., 2020).

The differential expression of milRNAs was determined according to the relative expression abundance of each miRNA in the MVm and MD2. When the |log2 (fold change)| of miRNA was ≥1 and value of *p*≤0.01, the expression was judged significantly different. Normalized expression level was calculated as mapped read count/total reads × 10^6^, with the value of *p* calculated this way:

$$P=\min\left\{\overset{k\leq y}{\underset{k=0}{\sum }}p\left(k/x\right),\overset{\infty }{\underset{k=y}{\sum }}p\left(k/x\right)\right\}$$

$$P\left(x/y\right)={\left(\frac{N2}{N1}\right)}^{y}\frac{\left(x+y\right)!}{x!y!{\left(1+\frac{N2}{N1}\right)}^{\left(x+y+1\right)}}$$

where *N*1 denotes the expression level of miRNAs in the wild-type strain (MVm), while *N*2 denotes the expression level of miRNAs in the *Vm*-*DCL2* deletion mutant (MD2); *x* represents all the miRNAs sequenced in the sample of the wild-type strain, and *y* represents all the miRNAs sequenced in the sample of the DCL2 mutant.

### Target Gene Identification of *Vm*-milRNAs by Degradome Sequencing

To verify the target genes of *Vm*-milRNAs, degradome sequencing was used. Samples of total RNA (each 20μg)—from the RNAs used for sRNA libraries construction—were used to construct the degradome sequencing library by following the protocols described previously (German et al., 2009). The specific degradome sequencing and data analysis methods were consistent with those used in a previous study (Xu et al., 2020).

### Sequence Alignment and Phylogenetic Analysis

Homologous protein sequences were searched by using the Blast function in NCBI Web site.^1^ For those proteins, their conserved domains were predicted by SMART and using the conserved domain database at NCBI.^2^ The alignment of multiple protein sequences was done with DNAMAN software, and the phylogenetic tree was built using the neighbor-joining method in MEGA 6 (bootstrap values were set as 1,000).

Co-expression of *Vm*-PC-3p-92107_6 and *Vm-VPS10* in *Nicotiana Benthamiana* Leaves

*Vm*-PC-3p-92107_6 and the predicted target region of *Vm*-*VPS10* were separately inserted into the empty pCAMBIA1302 vector with GFP as the reporter gene. Then, these two recombinant vectors were co-transformed into the same site of *N. benthamiana* leaves *via* the *Agrobacterium*-mediated transfection system (GV3101), as described by Weiberg et al. (2013). *Vm*-milR9 with no sequence similarity to *Vm*-PC-3p-92107_6 and mutated *Vm*-PC-3p-92107_6 were used as controls, respectively. Confocal images were taken at 48h post-*Agrobacterium* infiltration. The quantitative GFP intensity is proportional to the expression level of the candidae target gene. To further verify the expression of GFP, Western blot analysis was done using Anti-GFP (Sungene Biotech, Tianjin, China), with horseradish peroxidase-conjugated goat anti-mouse IgG (Cwbiotech, Beijing, China) used as the secondary antibody. The co-expression experiment was repeated twice, independently, for which all the primers can be found in Supplementary Table S1.

### Relative Expression of Pre-*Vm*-PC-3p-92107_6, *Vm*-PC-3p-92107_6 and *Vm-VPS10*

Total RNA was extracted using the miRcute Plant miRNA Isolation Kit (Tiangen, Beijing, China) according to the manufacturer’s instructions. For the expression of *Vm*-PC-3p-92107_6, it was detected with stem-loop qRT-PCR, as described by Xu et al. (2020). The first strand cDNA was synthesized by implementing the first strand cDNA synthesis of miRNA (Stem-Loop Method; Sangon Biotech, Shanghai, China) with the stem-loop RT primer, according to the manufacturer’s instructions. PCR amplification was performed using the *Vm*-PC-3p-92107_6-specific forward primers and universal reverse primers; small nuclear RNA U6 served as a control. To determine the transcript levels of precursor of *Vm*-PC-3p-92107_6 (pre-*Vm*-PC-3p-92107_6) and *Vm*-*VPS10*, a sequence-specific primer and oligo(dT) primer were used to carry out the reverse-transcription using the Revert Aid First Strand cDNA Synthesis Kit (Thermo Scientific, Waltham, MA, United States) according to the manufacturer’s instructions. The transcriptional level of pre-*Vm*-PC-3p-92107_6 and *Vm*-*VPS10* was analyzed by qRT-PCR, for which the glucose-6-phosphate dehydrogenase gene (*G6PDH*) served as the reference gene (Yin et al., 2013). The quantitative PCR was run on LightCycler 96 real-time PCR instrument (Roche, Basel, Switzerland). Relative expression levels were calculated by applying the 2^-ΔΔCt^ method (Shabalina and Koonin, 2008). All primers used in this study are in Supplementary Table S1.

### Generation of *Vm*-PC-3p-92107_6 Over-Expression Transformants, *Vm*-PC-3p-92107_6, *Vm-VPS10* Deletion Mutants, and the Complement Transformants

To over-express *Vm*-milRNA, the pre-*Vm*-PC-3p-92107_6 and its forward and reverse sequences of 200bp (31,247bp to 31,766bp in the *V. mali* wild-type strain genome contig 451) were amplified using Phusion High-fidelity DNA polymerase. The methodology used to derive the over-expression constructs in plasmid PDL2 is consistent with that described in Xu et al. (2020). The constructs were verified by sequencing and transformed into the wild-type strain. For the generation of mutated *Vm*-PC-3p-92107_6 (Mut-*Vm*-PC-3p-92107_6) constructs, the methods described by Xu et al. (2020) were also referred to and applied. All primers used for over-expression analyses are listed in Supplementary Table S1.

Double-joint PCR was used to build the deletion construction, and the specific construction process is consistent with already described by Yu et al. (2004). *Vm*-*VPS10* deletion mutants were generated using the strain Δ*Vm-Ku80*, which provided for highly enhanced target gene deletion efficiency but did not affect either vegetative growth or virulence (Xu et al., 2016). Four types of PCR detections were conducted to confirm that both *Vm*-PC-3p-92107_6 and *Vm*-*VPS10* were indeed deleted. To construct the complement vector, the gene was amplified, and then connected to the PDL2 vector that had been digested by the homologous recombination method. The positive vectors extracted from the competent state DH5α of *E. coli* were confirmed by sequencing, and then transformed into the corresponding deletion mutants. PCR was used detect the complement transformants. All primers used for genes deletion are given in Supplementary Table S1.

### Vegetative Growth and Pathogenicity Tests of Mutants

The vegetative growth and pathogenicity of gene deletion mutants and over-expression transformants were analyzed as previous described (Feng et al., 2017a). Briefly, fungal colony diameters were measured at 48h post-cultivation. The assay was independently performed three times, and each experiment had three replicates. Pathogenicity was tested using “Fuji” apple twigs, as described by Feng et al. (2017a). Lesion length was measured at 5days post-inoculation (dpi). The pathogenicity test was repeated three times, and each experiment had three replicates. Next, the significant difference in means was analyzed by a t-test (for two independent sample groups) and ANOVA (for three or more independent sample groups; at *p*≤0.05) in GraphPad Prism 6.0 software.

## Results

### Analysis of Small RNAs’ Abundance in MVm and MD2

The sequence data of MVm were deposited in NCBI (GEO accession no. GSM3757989) with the publication of a previous study (Xu et al., 2020). Referring to the previous study, 12,818,591 raw read sequences and 1,023,889 valid sequences were obtained in MVm, while in MD2 library, 11,565,746 raw read sequences were obtained and then after removing the repetitive sequence and junk sequences, valid was for only 593,982, which indicated the deletion of DCL2 could affect the generation of sRNAs in *V. mali* (Supplementary Table S2; Supplementary Figure S1A). Analyzing the length distribution of sRNAs revealed they had a consistent length of 19–24nt in both libraries (Supplementary Figure S1B); however, the abundance of sRNAs of a given same length differed. The sRNAs in MVm were mostly 20nt, 21nt, and 22nt, while those in MD2 were mainly composed of those 22nt and 23nt in length (Supplementary Figure S1C). Hence, we speculated that deletion of *Vm*-*DCL2* could somehow affect the generation of sRNA, but there may be an as of yet unknown complementary pathway that could compensate for part function of sRNAs generation.

### Isolation and Identification of DCL2-Associated siRNAs and milRNAs

**Figure 1:**
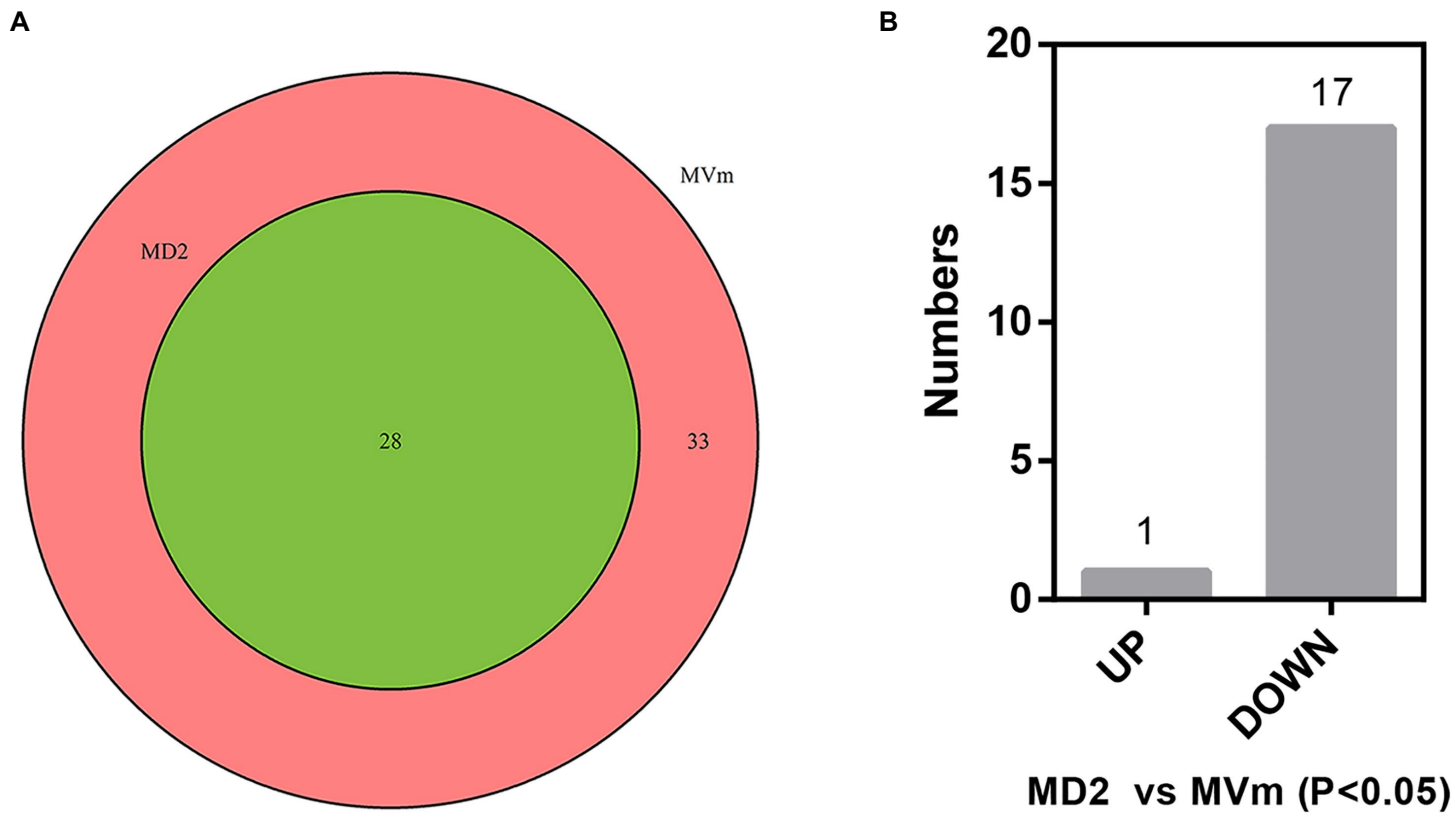
**(A)** Venn diagrams of detected miRNA-like RNAs (milRNAs). **(B)** The bar plot of differentially expressed milRNAs in the MD2 and MVm (P<0.05). MD2: Δ*Vm*-*DCL2*; MVm: WT.

The differential expression of siRNAs and milRNAs between the two libraries was first analyzed. Based on the sequencing results, 3,243 siRNAs were isolated and identified, of which 3,186 were not detected in MD2 (data not shown). Among these siRNAs, 25 were significantly upregulated in MD2 compared with MVm, including four siRNAs specifically expressed in MD2, and 15 were downregulated, with one siRNA not detected in MD2 (Supplementary Figure S2, Supplementary Table S6). Thus, the generation of siRNAs was greatly changed when *Vm*-*DCL2* was deleted. By comparing the difference in expression of milRNAs between MVm and MD2, 33 milRNAs were not detected in MD2 while 28 milRNAs were detected in both MVm and MD2 (Figure 1A, Supplementary Table S3). The statistical analysis revealed that 1 and 17 milRNAs, respectively, exhibited upregulated and downregulated expression in MD2 compared with MVm (*p*<0.05; Figure 1B). Importantly, 10 milRNAs were not detected in MD2, which were identified as being DCL2-dependent milRNAs (Table 1).

**Table 1:** DCL2-dependent milRNAs isolated from *V. mali.*

Index	miR_name	miR_sequence	up/down	p value (chi_square_2×2)
1	PC-3p-92107_6	TCGCAAGACTGTCCTGCTTGGCA	down	4.39E-11
2	PC-5p-92307_6	TAGAACTTAGAAGGTAGAGA	down	1.19E-03
3	PC-5p-128213_4	TAACTATAAGTAGAGCGCTC	down	6.48E-03
4	PC-3p-149663_4	TGATAGTTGGTTCGTGGTAGT	down	9.15E-03
5	PC-5p-257804_2	TAGATAGAACTTAAAAGGTAGA	down	1.30E-02
6	PC-3p-197143_3	TAGGGTTTATATTGTTAGAGA	down	1.84E-02
7	PC-5p-191126_3	TCGCTATAAGTCTTAGAACTAT	down	1.84E-02
8	PC-5p-352109_2	TTTAGTAGATTTATAAGCGT	down	1.84E-02
9	PC-5p-109000_5	AAGTATTTCGGATTATCGGGCG	down	2.20E-02
10	PC-5p-286351_2	AAAGTATTTCGGATTATCGGGCT	down	2.62E-02

### Target Prediction of DCL2-Associated milRNAs

The target genes of DCL2-associated milRNAs were identified by high-throughput degradome sequencing technology. In total, the target genes of 12 milRNAs were distinguishable. These target genes were annotated as follows: histidine kinase, serine/threonine-protein kinase, pectin esterase, glucose transport regulator, ATP-dependent RNA helicase, myosin, and AP-2 complex subunit, etc. They mainly participate in 35 different groups at the three categories of biological process, molecular function, and cell component (Supplementary Table S5). We found that PC-5p-164768_3, PC-5p-122130_5, PC-5p-136623_4, PC-5p-2366_295, PC-3p-3147_232, PC-3p-149663_4, PC-5p-128213_4, and PC-3p-4530_168 could target more than one gene. For example, PC-3p-4530_168 could target 31 genes, and these main target genes were associated with protein phosphatase, isocitrate dehydrogenase, phosphoglycerate mutase, etc. In stark contrast, PC-5p-31952_22, PC-3p-92107_6, PC-3p-355705_2, and PC-3p-15073_50 could only target one gene, such as Vm-PC-3p-92107_6 targeting VM1G_02763, the latter predicted to be *VPS10* (Table 2). Evidently, the regulatory network of milRNAs is very complex.

**Table 2:** Target genes of *Vm-*DCL2-associated milRNAs identified by degradome sequencing.

Small RNA	Target gene	Target gene annotation	p value (chis_quare2×2)
PC-5p-31952_22	VM1G_10292	pantoate--beta-alanine ligase (panC)	7.64E-03
PC-5p-164768_3	VM1G_00825	acetyl-CoA carboxylase/biotin carboxylase (cut6)	3.47E-03
	VM1G_02980	Stoml3	3.47E-03
PC-3p-92107_6	VM1G_02763	VPS10	0.00E+00
PC-5p-122130_5	VM1G_00814	PTK9 protein tyrosine kinase 9 (SFC1)	0.00E+00
	VM1G_03440	cytochrome c (cyc-1)	7.64E-03
PC-5p-136623_4	VM1G_03006	alcohol dehydrogenase (adh-1)	1.92E-28
	VM1G_04236	–	3.47E-03
PC-3p-355705_2	VM1G_06294	–	1.35E-11
PC-5p-2366_295	VM1G_02768	utp23	1.60E-03
	VM1G_08726	–	7.64E-03
PC-3p-15073_50	VM1G_00573	–	1.70E-02
PC-3p-3147_232	VM1G_03399	allantoicase (ANK3)	0.00E+00
	VM1G_00903	–	0.00E+00
	VM1G_07631	MICALL2	0.00E+00
	VM1G_01328	serine/threonine-protein kinase TTK/MPS1(MPS1)	0.00E+00
	VM1G_11095	citrate synthase (mcsA)	4.91E-76
	VM1G_09589	SDR3a	3.61E-38
	VM1G_10323	pep7	4.40E-08
	VM1G_06798	rplA	1.61E-04
	VM1G_06611	elongation factor EF-1 gamma subunit(CAM1)	1.61E-04
	VM1G_11372	–	3.47E-03
	VM1G_09340	–	7.64E-03
	VM1G_00064	high-affinity iron transporter (FTR1)	7.64E-03
PC-3p-149663_4	VM1G_09539	V-type H + -transporting ATPase subunit B (vma-2)	0.00E+00
	VM1G_04444	NADH dehydrogenase (ubiquinone) 1 alpha subcomplex 5 (nuo-32)	0.00E+00
PC-5p-128213_4	VM1G_10968	cyclopropane-fatty-acyl-phospholipid synthase	5.15E-03
	VM1G_10967	cyclopropane-fatty-acyl-phospholipid synthase(ERG6)	5.15E-03
PC-3p-4530_168	VM1G_11253	impact	0.00E+00
	VM1G_07261	impact	0.00E+00
	VM1G_04770	DnaJ homolog, subfamily C, member 2 (zuo1)	0.00E+00
	VM1G_10029	protein phosphatase (ptc2)	0.00E+00
	VM1G_02181	isocitrate dehydrogenase (NAD+; IDH1)	3.87E-59
	VM1G_02403	–	3.46E-20
	VM1G_07697	phosphoglycerate mutase (gpmI)	1.84E-14
	VM1G_07984	mog1	3.46E-13
	VM1G_04561	fatty acid synthase subunit alpha, fungi type (FAS2)	3.11E-12
	VM1G_11912	–	3.11E-12
	VM1G_04952	protein phosphatase (Pak4)	3.11E-12
	VM1G_07199	H/ACA ribonucleoprotein complex subunit 4 (cbf5)	2.54E-10
	VM1G_08346	–	2.31E-09
	VM1G_03726	TOXD	2.31E-09
	VM1G_04952	protein phosphatase (Pak4)	4.40E-08
	VM1G_08663	VPS73	4.40E-08
	VM1G_04421	adenosylmethionine-8-amino-7-oxononanoate aminotransferase (BIO3-BIO1)	1.93E-07
	VM1G_10102	MAST3	4.05E-07
	VM1G_10102	MAST3	3.76E-06
	VM1G_06643	small subunit ribosomal protein S3Ae (RPS1)	7.94E-06
	VM1G_03002	–	7.94E-06
	VM1G_00061	–	7.54E-05
	VM1G_09785	fluG	1.61E-04
	VM1G_03964	chaperonin GroES (hsp10)	1.61E-04
	VM1G_06032	mdm28	7.38E-04
	VM1G_07984	mog1	7.38E-04
	VM1G_05865	Rho family, other (rac-2)	7.38E-04
	VM1G_02634	inorganic pyrophosphatase (ipp-1)	3.47E-03
	VM1G_11151	–	7.64E-03
	VM1G_01547	argininosuccinate synthase (ARG1)	7.64E-03
	VM1G_04853	ATP synthase mitochondrial F1 complex assembly factor 2 (ATP12)	7.64E-03
	VM1G_01644	DnaJ homolog, subfamily A, member 2 (mas5)	7.64E-03

### *Vm*-PC-3p-92107_6 Is a DCL2-Dependent milRNA

**Figure 2:**
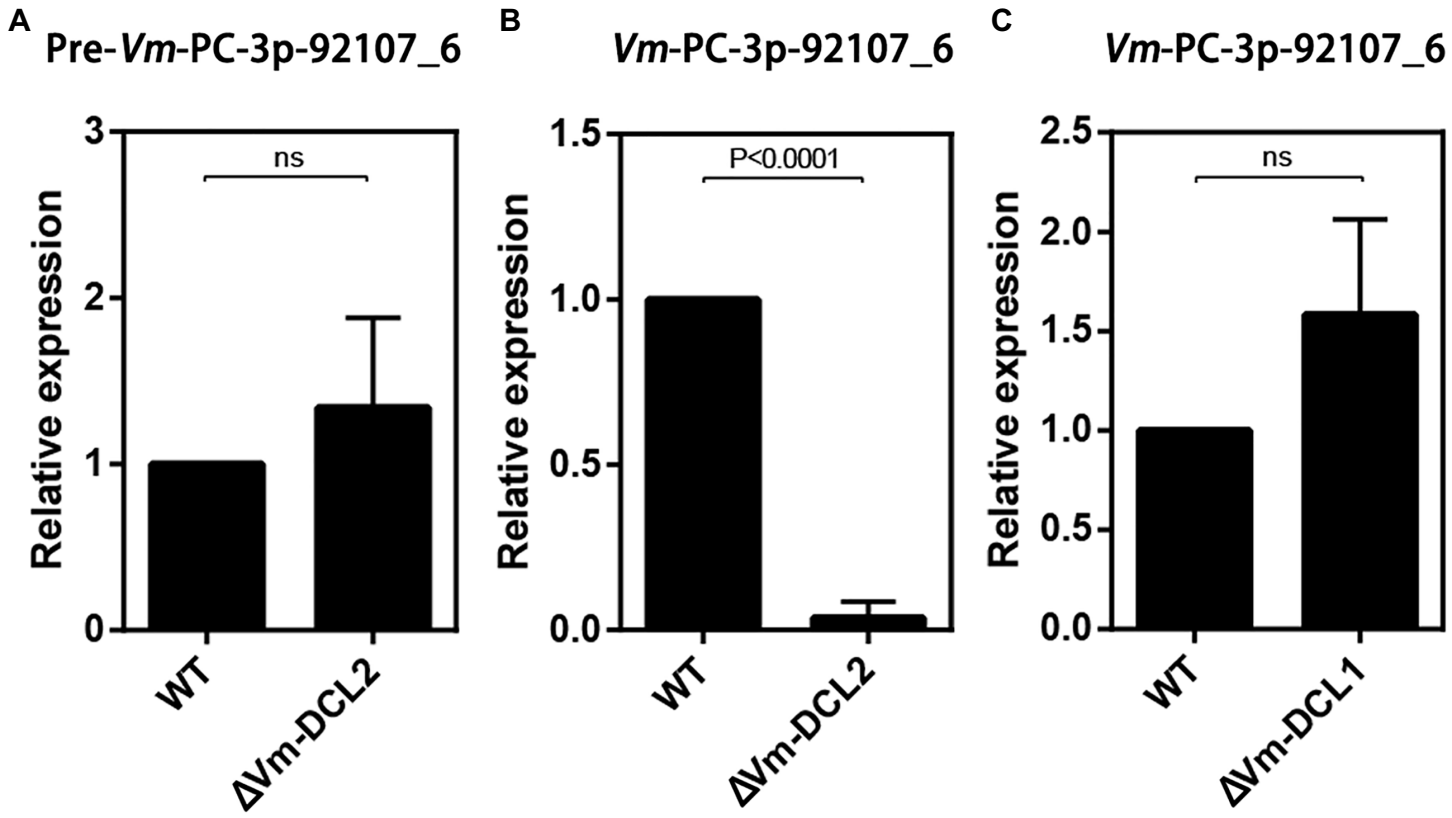
Relative expressions of pre-*Vm*-PC-3p-92107_6 and *Vm*-PC-3p-92107_6 by quantitative real-time PCR. **(A)** Relative expression of pre-*Vm*-PC-3p-92107_6 in *Vm*-*DCL2* deleted mutant (Δ*Vm*-*DCL2*). *Vm*-*G6PDH* was selected to reference gene and the relative expression level of pre-*Vm*-PC-3p-92107_6 in WT was set to 1 by the 2^−ΔΔCt^ method. **(B)** Relative expression of *Vm*-PC-3p-92107_6 in *Vm*-*DCL2* deleted mutant (Δ*Vm*-*DCL2*). *Vm*-U6 was selected to reference gene and the relative expression level of *Vm*-PC-3p-92107_6 in WT was set to 1. **(C)** Relative expression of *Vm*-PC-3p-92107_6 in *Vm*-*DCL1* deleted mutant (Δ*Vm*-*DCL1*). *Vm*-U6 was selected to reference gene and the relative expression level of *Vm*-PC-3p-92107_6 in WT was set to 1.

Based on the sequencing results, we found that *Vm*-PC-3p-92107_6 could not be detected in MD2. Accordingly, we speculated that *Vm*-PC-3p-92107_6 is a DCL2-dependent milRNA. To test this hypothesis, the relative expression levels of pre-*Vm*-PC-3p-92107_6 (*Vm*-PC-3p-92107_6-P) and *Vm*-PC-3p-92107_6 were detected by qRT-PCR in the wild type and DCL2 mutant (Δ*Vm*-*DCL2*), respectively. The expression of pre-*Vm*-PC-3p-92107_6 showed no significant difference between the wild type and Δ*Vm*-*DCL2*, whereas that of *Vm*-PC-3p-92107_6 was barely detected in Δ*Vm*-*DCL2* (Figures 2A,B). Meanwhile, the expression of *Vm*-PC-3p-92107_6 was detectable in the *Vm*-*DCL1* mutant (Δ*Vm*-*DCL1*) but at a similar level as in the wild type; likewise, no significant differences were found in the expression level of *Vm*-PC-3p-92107_6 in Δ*Vm*-*DCL1* relative to the wild type (Figure 2C). These results further proved that *Vm*-PC-3p-92107_6 is specifically dependent on *Vm*-*DCL2* in *V. mali*.

### *Vm*-PC-3p-92107_6 Played Important Roles in Vegetative Growth and Pathogenicity

**Figure 3:**
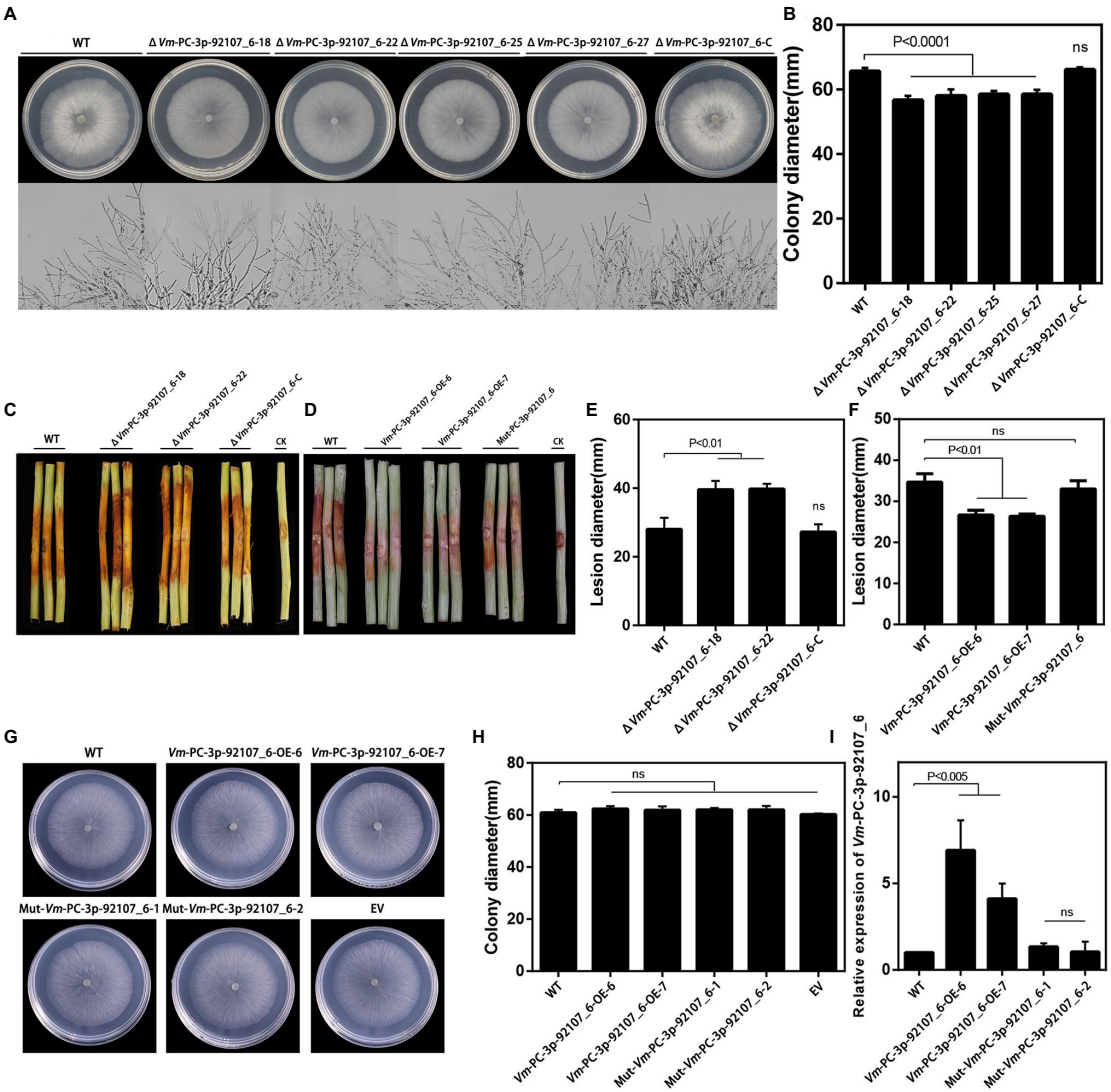
Phenotypical analysis of *Vm*-PC-3p-92107_6 deleted mutants (Δ*Vm*-PC-3p-92107_6), complement transformants (Δ*Vm*-PC-3p-92107_6-C), over-expression transformants (*Vm*-PC-3p-92107_6-OE), and the wild type (WT). **(A)** and **(B)** Colony morphology of WT, *Vm*-PC-3p-92107_6 deleted mutants (Δ*Vm*-PC-3p-92107_6–18, Δ*Vm*-PC-3p-92107_6–22, Δ*Vm*-PC-3p-92107_6–25, and Δ*Vm*-PC-3p-92107_6–27), and complement transformants (Δ*Vm*-PC-3p-92107_6-C) after 48h (hr) incubation in PDA at 25°C. Related colony diameters were measured after 48h incubation. Data represent mean±SD. The experiment was repeated three times, each time with three plates. **(C)** and **(E)** Pathogenicity test of WT, Δ*Vm*-PC-3p-92107_6–18, Δ*Vm*-PC-3p-92107_6–22, and Δ*Vm*-PC-3p-92107_6-C after 5days post-inoculation. Three representative diseased twigs are shown. The pathogenicity test was independently repeated three times, each time with six replicates. CK represents a negative control. Data represent mean±SD. **(D)** and **(F)** Pathogenicity test of WT, *Vm*-PC-3p-92107_6-OE-6, *Vm*-PC-3p-92107_6-OE-7, and Mut-*Vm*-PC-3P-92107_6 after 5dpi. Three representative diseased twigs are shown. The pathogenicity test was independently repeated three times, each time with six replicates. CK represents a negative control. Data represent mean±SD. **(G)** and **(H)** Colony morphology of WT, *Vm*-PC-3p-92107_6 over-expression transformants (*Vm*-PC-3p-92107_6-OE-6 and *Vm*-PC-3p-92107_6-OE-7), mutated *Vm*-PC-3p-92107_6 (Mut-*Vm*-PC-3P-92107_6–1 and Mut-*Vm*-PC-3P-92107_6–2), and empty vector transformant (EV) after 48h incubation in PDA at 25°C. Related colony diameters were measured after 48h incubation. Data represent mean±SD. The experiment was repeated three times, each time with three plates. **(I)** The relative expression level of *Vm*-PC-3p-92107_6 in WT, *Vm*-PC-3p-92107_6 over-expression transformants (*Vm*-PC-3p-92107_6-OE-6 and *Vm*-PC-3p-92107_6-OE-7), and mutated *Vm*-PC-3p-92107_6 (Mut-*Vm*-PC-3P-92107_6-1 and Mut-*Vm*-PC-3P-92107_6-2). *Vm*-U6 was selected to reference, the relative expression level of WT is set to 1 as control group using the 2^-ΔΔCt^ method. Data represent mean ± SD.

To clarify the function of *Vm*-PC-3p-92107_6, the *Vm*-PC-3p-92107_6 deletion mutants (Δ*Vm*-PC-3p-92107_6) and complement transformants (Δ*Vm*-PC-3p-92107_6-C) were constructed (Supplementary Figure S3, S4). Compared with the wild type, the colony diameter of Δ*Vm*-PC-3p-92107_6 was significantly reduced, yet there was little difference in either their colony morphology or density of aerial hyphae (Figures 3A,B). Moreover, compared with the wild type, the pathogenicity of Δ*Vm*-PC-3p-92107_6 was significantly augmented, but after the *Vm*-PC-3p-92107_6 complementation, it was similar to the wild type (Figures 3C,E).

To further confirm the function of *Vm*-PC-3p-92107_6, the *Vm*-PC-3p-92107_6 over-expression transformants (*Vm*-PC-3p-92107_6-OE) and the Mut-*Vm*-PC-3p-92107_6 mutants (Mut-*Vm*-PC-3p-92107_6) were also constructed (Supplementary Figure S5). In comparison with the wild type, the colony diameter of *Vm*-PC-3p-92107_6-OE, Mut-*Vm*-PC-3p-92107_6, and EV showed no significant differences (Figures 3G,H). More importantly, the pathogenicity of *Vm*-PC-3p-92107_6-OE was significantly reduced, whereas it was not significantly different between Mut-*Vm*-PC-3p-92107_6 and EV (Figures 3D,F). This confirmed that *Vm*-PC-3p-92107_6 could regulate the vegetative growth and pathogenicity of *V. mali*.

### *Vm-VPS10* Was Identified as the Target of *Vm*-PC-3p-92107_6

**Figure 4:**
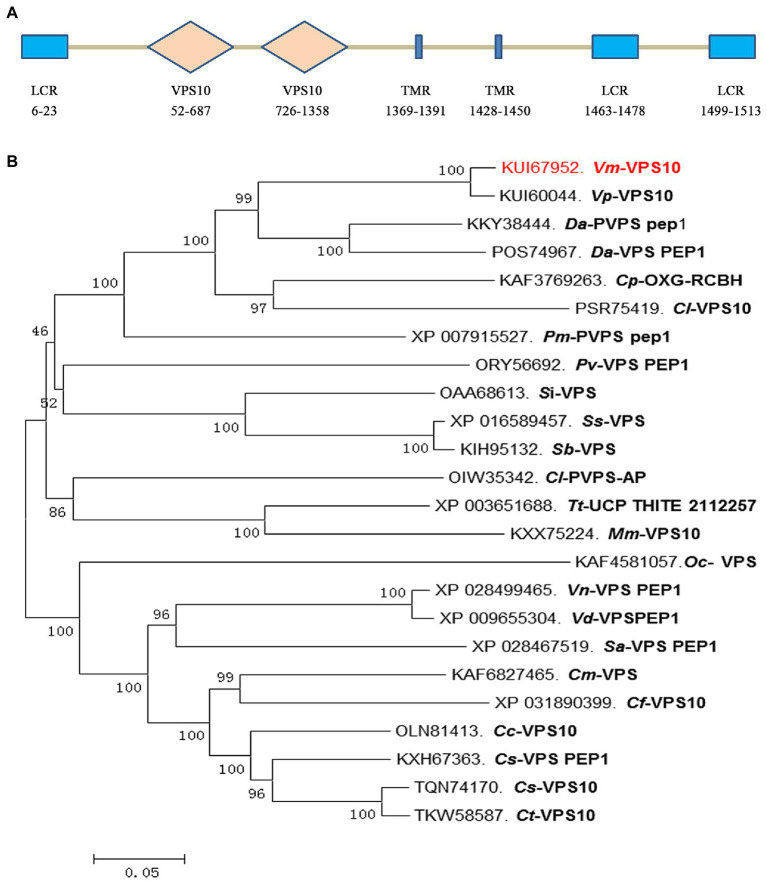
Protein sequence characterization and phylogeny analysis of the *Vm*-*VPS10*. **(A)** The typical domains of *Vm*-*VPS10*. *Vm*-*VPS10* contains three conserved domains, including low complexity region (LCR), *VPS10*, and transmembrane region (TMR). *VPS10* is the core domain, which is a receptor domain, and TMR is an important transmembrane helix region. The numbers below the structure indicate the amino acid sites of each domain. **(B)** The phylogenetic tree was constructed with neighbor-joining method using MEGA 6. Bootstrap values were set as 1,000. *Vm*-*VPS10* is highlighted in red. *Vm*, *Valsa mali; Vp, Valsa pyri; Da, Diaporthe ampelina; Dh, Diaporthe helianthi; Cp, Cryphonectria parasitica; Cl, Coniella lustricola; Pm, Phaeoacremonium minimum; Pv, Pseudomassariella vexata; Si, Sporothrix insectorum; Sc, Sporothrix schenckii; Sb, Sporothrix brasiliensis; Tt, Thermothielavioides terrestris; Mm, Madurella mycetomatis; Oc, Ophiocordyceps camponoti-floridani; Vn, Verticillium nonalfalfae; Sa, Sodiomyces alkalinus; Cm, Colletotrichum musicola; Cf, Colletotrichum fructicola; Cc, Colletotrichum chlorophyte; Cs, Colletotrichum salicis; and Ct, Colletotrichum tanaceti.*

Based on the degradome sequencing results, only the transcript VM1G_02763 was identified to be the target of *Vm*-PC-3p-92107_6. The bioinformatics analysis indicated that VM1G_02763 encoded a protein with three low complexity regions, two *VPS10* regions, and two transmembrane regions, which we designated here as *Vm*-*VPS10* (Figure 4A). The phylogeny of vacuolar protein sorting protein (VPS) between *V. mali* and other 23 filamentous fungi was investigated by constructing a neighbor-joining phylogenetic tree. This revealed that *Vm*-*VPS10* (KUI67952) is highly homologous to *VPS10* of *Valsa pyri* (KUI60044; Figure 4B).

**Figure 5:**
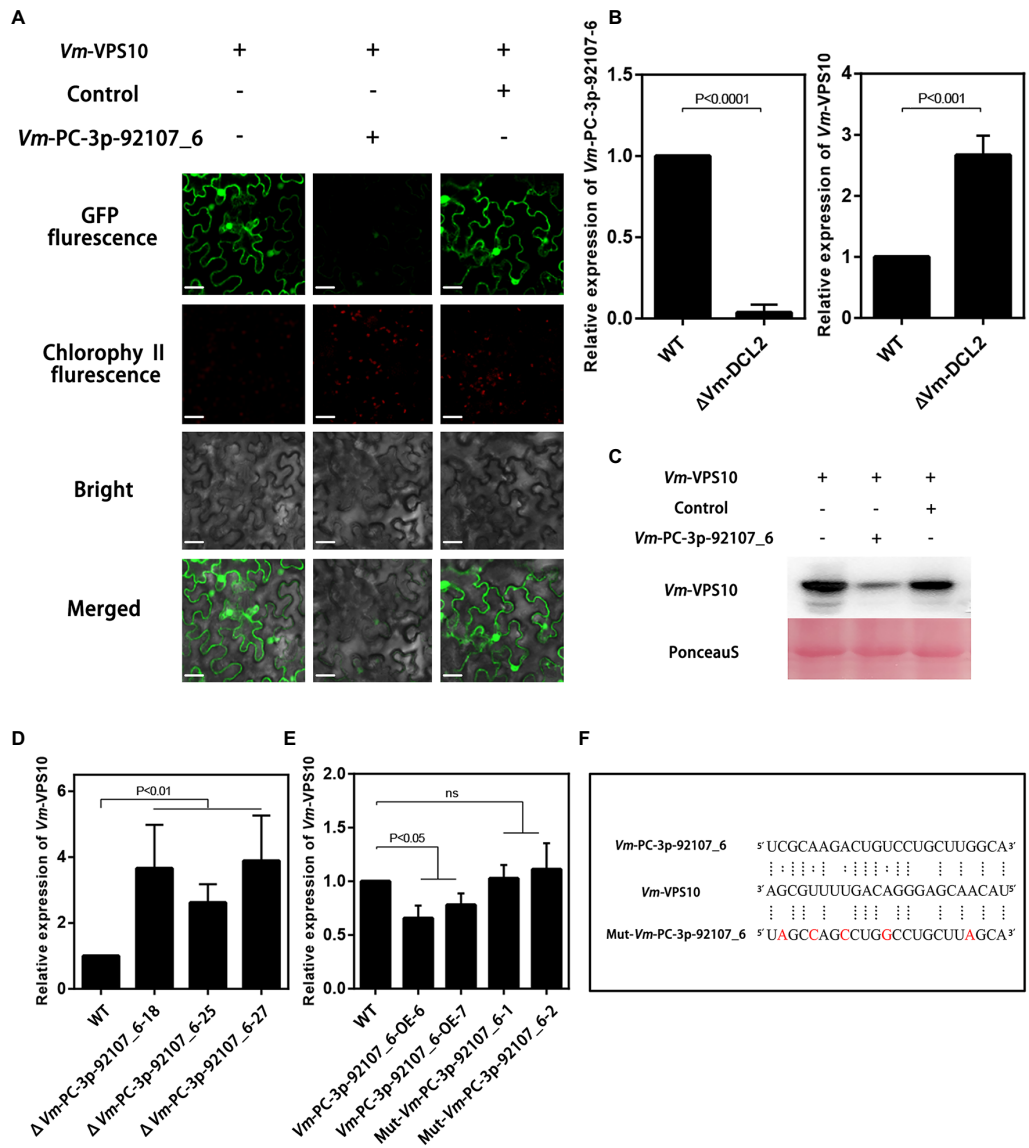
The regulatory relationship analysis between *Vm*-PC-3p-92107_6 and *Vm*-*VPS10*. **(A)** *Vm*-*VPS10* expression vectors and *Vm*-PC-3p-92107_6-OE expression vectors were constructed, respectively. *Vm*-milR9 with no sequence similarity was used as the control. Confocal imaging was performed 48h after *Agrobacterium* infiltration. **(B)** The relative expression of *Vm*-PC-3p-92107_6 and *Vm*-*VPS10* in WT and *Vm*-*DCL2* deleted mutants (Δ*Vm*-*DCL2*). *Vm*-U6 and *Vm*-*G6PDH* were selected to reference gene using 2^-ΔΔCt^ method. And the relative expressions of *Vm*-PC-3p-92107_6 and *Vm*-*VPS10* in WT were set to 1 which is taken as control. **(C)** Western blot analysis of eGFP-*Vm*-*VPS10*. Anti-GFP antibodies were used for analysis. The co-expression experiment was repeated twice and similar results were obtained. **(D)** The relative expression of *Vm*-*VPS10* in WT and *Vm*-PC-3p-92107_6 deleted mutants (Δ*Vm*-PC-3p-92107_6–18, Δ*Vm*-PC-3p-92107_6–25, and Δ*Vm*-PC-3p-92107_6–27). **(E)** The relative expression of *Vm*-*VPS10* in WT and over-expression transformants of *Vm*-PC-3p-92107_6 (*Vm*-PC-3p-92107_6-OE-6 and *Vm*-PC-3p-92107_6-OE-7), and mutated *Vm*-PC-3p-92107_6 (Mut-*Vm*-PC-3p-92107_6-1 and Mut-*Vm*-PC-3p-92107_6-2). **(F)** Sequence alignment of *Vm*-*VPS10* with *Vm*-PC-3p-92107_6 and mutated *Vm*-PC-3p-92107_6 (Mut-Vm-PC-3p-92107_6).

To further verify the regulatory relationship between *Vm*-PC-3p-92107_6 and *Vm*-*VPS10*, the expression vectors were successfully constructed and co-transformed into *N. benthamiana* leaves (Supplementary Figure S6). These results clearly showed green fluorescence visible on the leaves injected with *Vm*-*VPS10* alone. However, when *Vm*-PC-3p-92107_6 and *Vm*-*VPS10* were co-expressed, the intensity of green fluorescence was significantly diminished and the controls indistinguishable from *Vm*-*VPS10* alone. Meanwhile, when *Vm*-milR9, which has no sequence similarity with *Vm*-PC-3P-92107_6, was co-expressed with *Vm*-*VPS10*, the intensity of green fluorescence was similar with *Vm*-*VPS10* alone. The result of Mut-*Vm*-PC-3p-92107_6 also showed similar result (Figure 5A; Supplementary Figure S7). Further, the expression of GFP in the tissues co-expressing *Vm*-PC-3p-92107_6 and *Vm*-*VPS10* was significantly reduced in the Western blot analysis (Figure 5C). Collectively, these results indicated that *Vm*-PC-3p-92107_6 could suppress the expression of *Vm*-*VPS10*.

**Figure 6:**
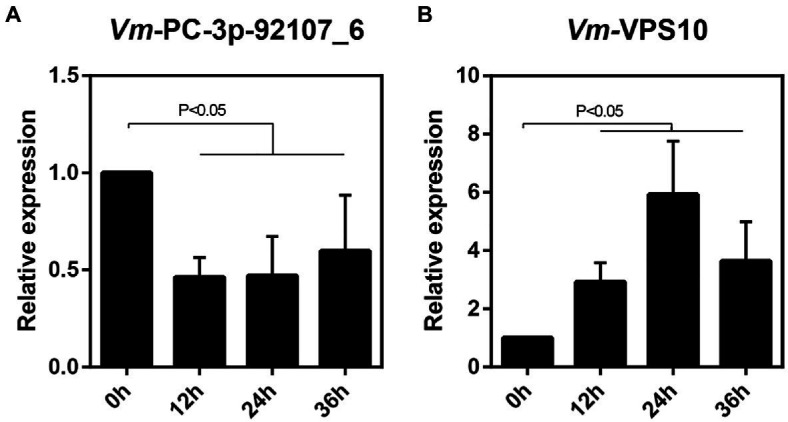
The relative expression pattern of Vm-PC-3p-92107_6 (A) and Vm-VPS10 (B) during V. mali infection.

Next, the relative expression levels of *Vm*-*VPS10* and *Vm*-PC-3p-92107_6 were determined by qRT-PCR in the wild type and Δ*Vm*-*DCL2*, respectively. When compared with the wild type, the expression level of *Vm*-*VPS10* in Δ*Vm*-*DCL2* was significantly upregulated (Figure 5B). Meanwhile, the relative expression of *Vm*-*VPS10* was also detected in both Δ*Vm*-PC-3p-92107_6 and *Vm*-PC-3p-92107_6-OE mutants. Compared with the wild type, the relative expression of *Vm*-*VPS10* was significantly increased in Δ*Vm*-PC-3p-92107_6, whereas it was significantly decreased in *Vm*-PC-3p-92107_6-OE (Figures 5D,E). Moreover, during the infection progress of *V. mali*, *Vm*-PC-3p-92107_6 was significantly downregulated at 12, 24, and 36 hpi (Figure 6A), yet *Vm*-*VPS10* had enhanced transcript levels during infection (Figure 6B). These results provided compelling evidence that the expression of *Vm*-*VPS10* could be regulated by *Vm*-PC-3p-92107_6.

### *Vm-VPS10* Contributed to Vegetative Growth and Pathogenicity

**Figure 7:**
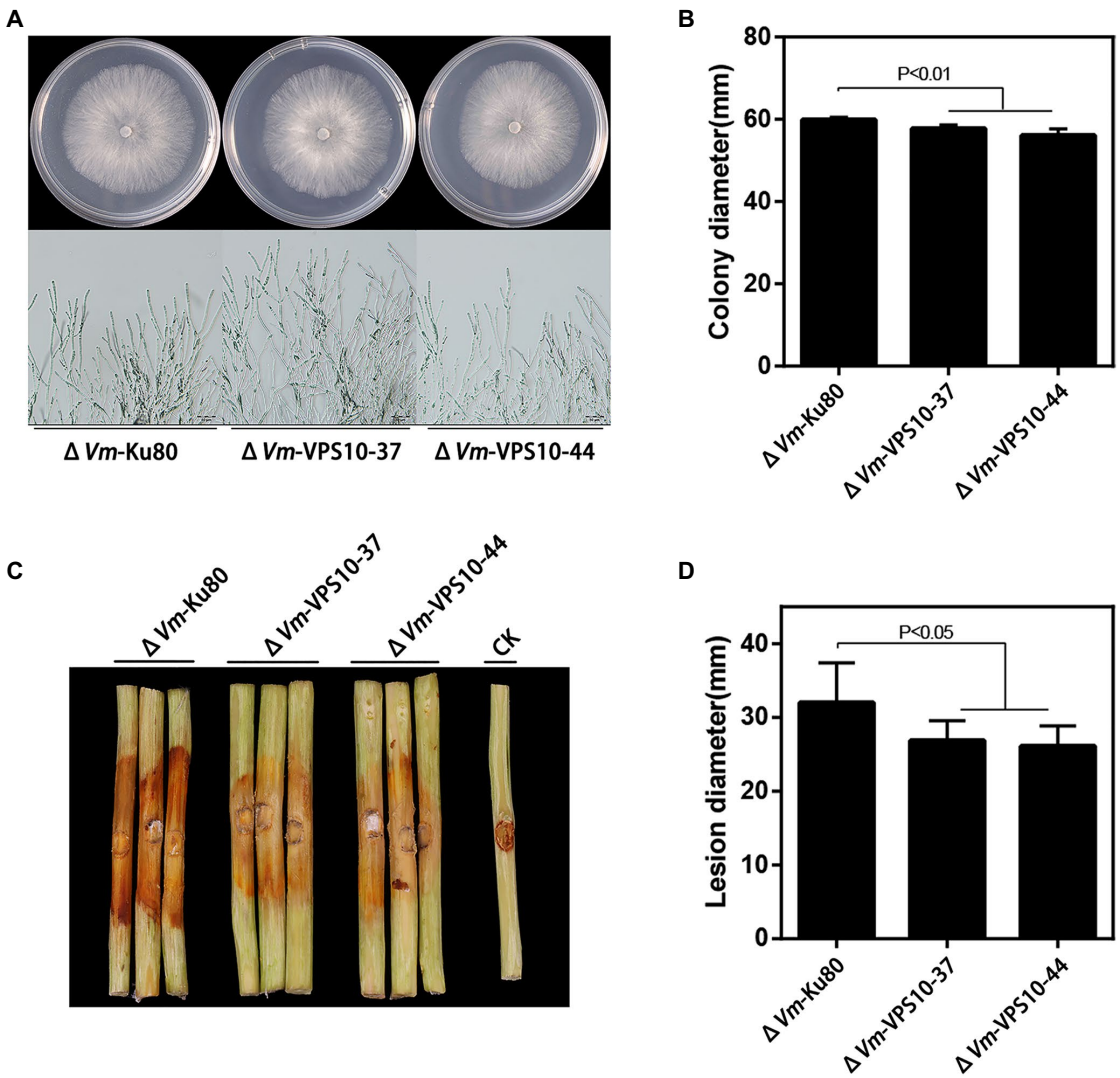
Phenotypical analysis of *Vm*-*VPS10* deleted mutants (Δ*Vm*-*VPS10*) and Δ*Vm*-*Ku80*. **(A)** Colony morphology of Δ*Vm*-*Ku80* and *Vm*-*VPS10* deleted mutants (Δ*Vm*-*VPS10*-37 and Δ*Vm*-*VPS10*-44) after 48h incubation in PDA at 25°C. **(B)** Colony diameters of Δ*Vm*-*Ku80* and Δ*Vm*-*VPS10*-37, Δ*Vm*-*VPS10*-44 after 48h incubation. Data represent mean ± SD. **(C)** and **(D)** Pathogenicity test of Δ*Vm*-*Ku80* andΔ*Vm*-*VPS10*-37, Δ*Vm*-*VPS10*-44 after 5dpi. Three representative diseased twigs are shown. The pathogenicity test was independently repeated three times, each time with six replicates. CK represents a negative control. Data represent mean ± SD.

Finally, to explore the function of *Vm*-*VPS10*, a gene deletion mutant (Δ*Vm*-*VPS10*) was constructed using Δ*VmKu80* (Supplementary Figure S8). Compared with Δ*VmKu80*, the colony diameter of Δ*Vm*-*VPS10* was significantly reduced, but both the density and morphology of airborne mycelia were not affected (Figures 7A,B). Notably, the pathogenicity was significantly lower for Δ*Vm*-*VPS10* than Δ*VmKu80* (Figures 7C,D).

## Discussion

As important non-coding RNA regulators, sRNAs play key roles in many biological processes, such as development regulation, transposon inhibition, environmental response, and host–pathogen interactions (Mallory and Vaucheret, 2006; Inui et al., 2010). As their key switch, Dicer proteins play crucial roles in the generation process of sRNAs (Nicolas et al., 2010). Still, the generation of sRNA is a more complex phenomenon in fungi. Since the first discovery of siRNA in *N. crassa* (Cogoni and Macino, 1999), research on the isolation of fungi sRNAs has expanded immensely, especially concerning the generation mechanism and functioning of sRNAs. In *N. crassa*, there are at least four pathways by which milRNAs are generated, including those that are DCL-dependent and DCL-independent (Lee et al., 2010). In *F. graminearum*, the generation of milRNAs was confirmed to be associated with *FgAGO1* and *FgDicer2* (Chen et al., 2015), and the Dicer-independent pathway to generate milRNA also exists in *M. circinelloides* (Nicolas and Ruiz-Vazquez, 2013). In *V. mali*, it has been shown that *Vm*-*DCL1* and *Vm*-*DCL2* deletion mutants could significantly reduce the production of sRNAs (Feng et al., 2017a). In our study, the differences in sRNAs between the wild type and *Vm*-*DCL2* deletion mutant were investigated. The results supported the view that the deletion of *Vm*-*DCL2* could affect the generation of sRNAs. Going further, we speculated another complementary pathway might exist that compensates for part function of sRNA generation when *Vm*-*DCL2* was deleted.

The miRNAs in plants and animals are crucially involved in their growth, development, reproduction, and responses to biotic and abiotic stresses, by inhibiting their corresponding target genes *via* transcriptional inhibition, mRNA cutting, or translation inhibition (Carrington and Ambros, 2003; Ghildiyal and Zamore, 2009; D’Ario et al., 2017; Wang et al., 2017; Song et al., 2019; Wang and Galili, 2019). However, the functions and mechanisms of milRNAs in fungi are still largely unknown. Some studies have shown that gene expression in fungi could be regulated at the post-transcriptional level to enable their adaption to various environments (Tan and Oliver, 2017). Similarly, the expression of many virulence genes could also be regulated by sRNAs in pathogenic fungi. For example, the expression of pathogenicity-related genes in *Magnaporthe grisea* can be regulated by sRNAs to affect its growth, development, and pathogenicity of this fungus (Nunes et al., 2011; Raman et al., 2013), with similar results reportedly found in *Trichoderma reesei* and *V. dahliae* (Kang et al., 2013; Jin et al., 2019). In *V. mali*, *Vm*-milR16 was identified to adaptively regulate the expression of its virulence genes *VmSNF1*, *VmDODA*, and *VmHy1*, thereby contributing to the infection ability of *V. mali* (Xu et al., 2020). In the present study, a DCL2-dependent milRNA, *Vm*-PC-3p-92107_6, was clearly associated with fungal vegetative growth and pathogenicity by regulating the expression of a vacuolar protein sorting protein (*Vm*-*VPS10*). VPS was first isolated in *Saccharomyces cerevisiae*; it encodes a type I transmembrane receptor protein, which is sequentially aggregated by binding to soluble proteins in cells (Cooper and Stevens, 1996). As such, it can figure prominently in protein transport, in which most proteins entering the vacuole are transported from the endoplasmic reticulum to the Golgi complex along with secreted proteins and are then sorted by sorting apparatus from other secretory traffic in the late Golgi lumen for specific transport to the vacuole. In filamentous fungi, the involvement of *VPS74* of *F. gramineae* in mycelia growth, conidia production, sexual reproduction, toxin production, and pathogenic process has been confirmed (Kim et al., 2015). In our study, functional analysis of *Vm*-*VPS10* suggested it could influence the growth and pathogenicity of *V. mali* that are regulated by milRNAs at the post-transcriptional level, which further broadens our understanding of the regulatory mechanism underpinning *VPS10* activity. Although the exact regulation mechanism is still unknown, we do know that *Vm*-*VPS10*’s expression could be suppressed by *Vm*-PC-3p-92107_6 to some extent. As mentioned above, the generation mechanism of sRNAs in fungi is more complex than that in plants and animals. Thus, we speculate the action mechanism of fungal sRNAs may also be more intricate, that is, to say, there may be more than three inhibition pathways by which fungal sRNAs impact their target genes.

Overall then, this study proved that DCL2-dependent milRNAs exist in *V. mali*. Among them, as a representative milRNA, *Vm*-PC-3p-92107_6 was confirmed to be involved in both vegetative growth and pathogenicity by regulating the expression of *Vm*-*VPS10*. This study enhances our understanding of the pathogenic mechanism of *V. mali* and helps to pave the way for fully revealing the generation and regulation mechanisms of fungal sRNA. Nonetheless, the functions of other DCL2-dependent sRNAs, especially their relationships, are still unclear and need further study.

## Data Availability Statement

The datasets presented in this study can be found in online repositories. The names of the repository/repositories and accession number(s) can be found at NCBI GEO, accession no: GSM3757989.

## Author Contributions

FG and HF conceived and designed the work. FG, JL, MX, and GZ performed the experiments. FG and JL analyzed and interpreted the data. JL drafted the manuscript. HF and LH critically revised the manuscript for intellectual content. All authors have read and agreed to the published version of the manuscript.

## Funding

This research was supported by the Natural Science Basis Research Plan in Shaanxi Province of China (no. 2019JM-418), the New Star of Youth Science and Technology of Shaanxi Province (no. 2021KJXX-10), and the China Postdoctoral Science Foundation funded project (no. 2015M580883).

## Conflict of Interest

The authors declare that the research was conducted in the absence of any commercial or financial relationships that could be construed as a potential conflict of interest.

## Publisher’s Note

All claims expressed in this article are solely those of the authors and do not necessarily represent those of their affiliated organizations, or those of the publisher, the editors and the reviewers. Any product that may be evaluated in this article, or claim that may be made by its manufacturer, is not guaranteed or endorsed by the publisher.

## Footnotes

^1^ https://blast.ncbi.nlm.nih.gov/Blast.cgi?PROGRAM=blastp&PAGE_TYPE=BlastSearch&LINK_LOC=blasthome

^2^ http://www.ncbi.nlm.nih.gov/Structure/cdd/wrpsb.cgi
