# Supplementary material for: A Novel DCL2-Dependent Micro-Like RNA Vm-PC-3p-92107_6 Affects Pathogenicity by Regulating the Expression of Vm-VPS10 in Valsa mali
Source: Front Microbiol. 2021 Oct 1;12:721399. doi: 10.3389/fmicb.2021.721399 (PMC8575173; doi:10.3389/fmicb.2021.721399)
Supplement: Supplementary file 1 [file Presentation_1.ZIP › Presentation_1.ZIP/Supplementary_Figures.docx]

Supplementary Material

## Supplementary Figures


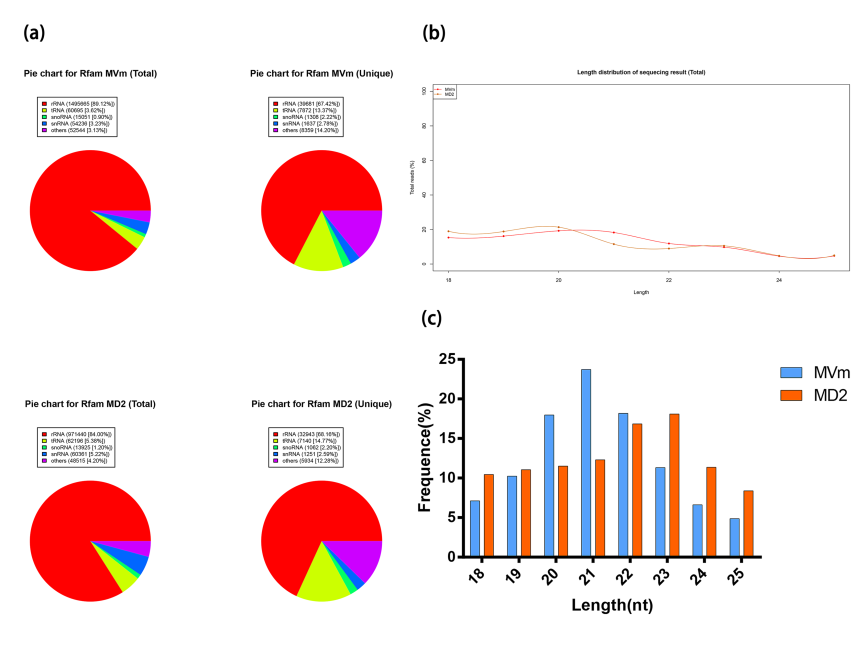


**Supplementary Figure 1.** Small RNAs sequencing data analysis. (a) Rfam sequence category of small RNAs in the wild-type (WT) and *Vm*-DCL2 mutant. (b)(c) The length distribution frequency of unique sequences in M*Vm* and MD2.


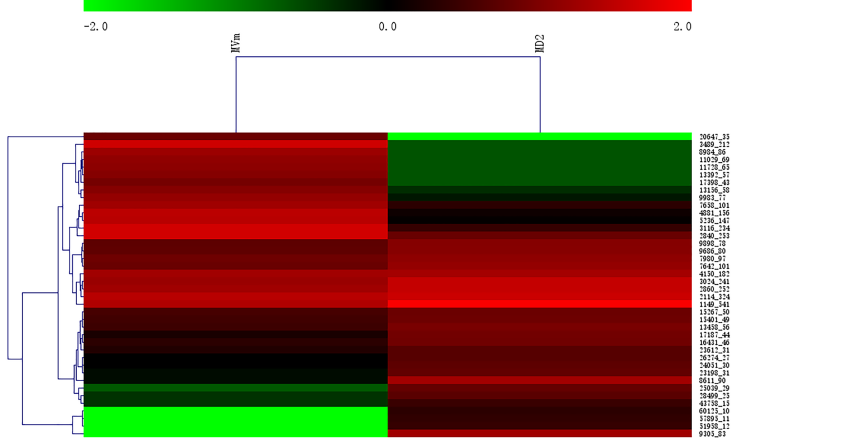


**Supplementary Figure 2.** Heat map of differentially expressed siRNAs

**
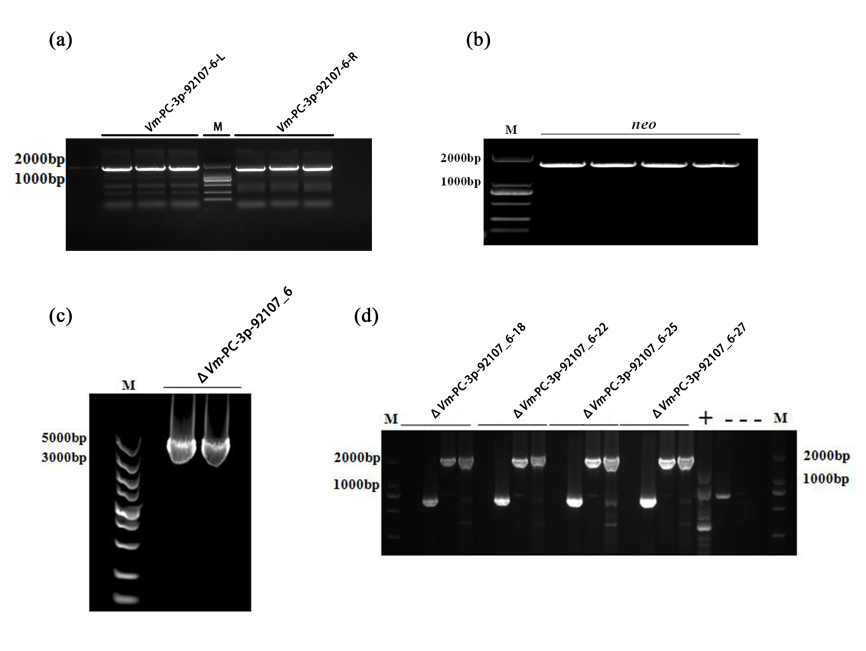
**

**Supplementary Figure 3.** Construction of *Vm*-PC-3p-92107_6 deleted mutants. (a) Detection of the upstream and downstream fragment of *Vm*-PC-3p-92107_6. (b) PCR amplification of Neo. (c) Construction of deleted cassette. (d) PCR analysis of deletion mutants by using the four types of primers (*Vm*-PC-3p-92107_6-5F/6R, *Vm*-PC-3p-92107_6-G850F/G852R, *Vm*-PC-3p-92107_6-7F/G855R, G856F/*Vm*-PC-3p-92107_6-8R). (First lane) Gene specific primers 5F/6R designed at the inner part of *Vm*-PC-3p-92107_6 were used to verify the deletion of *Vm*-PC-3p-92107_6. (Second lanes) Primer pair G852-F/G850-R designed at the inner core of NEO was used to verify the insertion of NEO. Two pairs of combined primers gene specific 7F/G855-R (third lane) and G856-F/gene specific 8R (fourth lane) were used to ascertain the homologous recombination upstream and downstream. Genomic DNA of WT and sterile double-distilled H_2_O were used as positive and negative control, respectively.


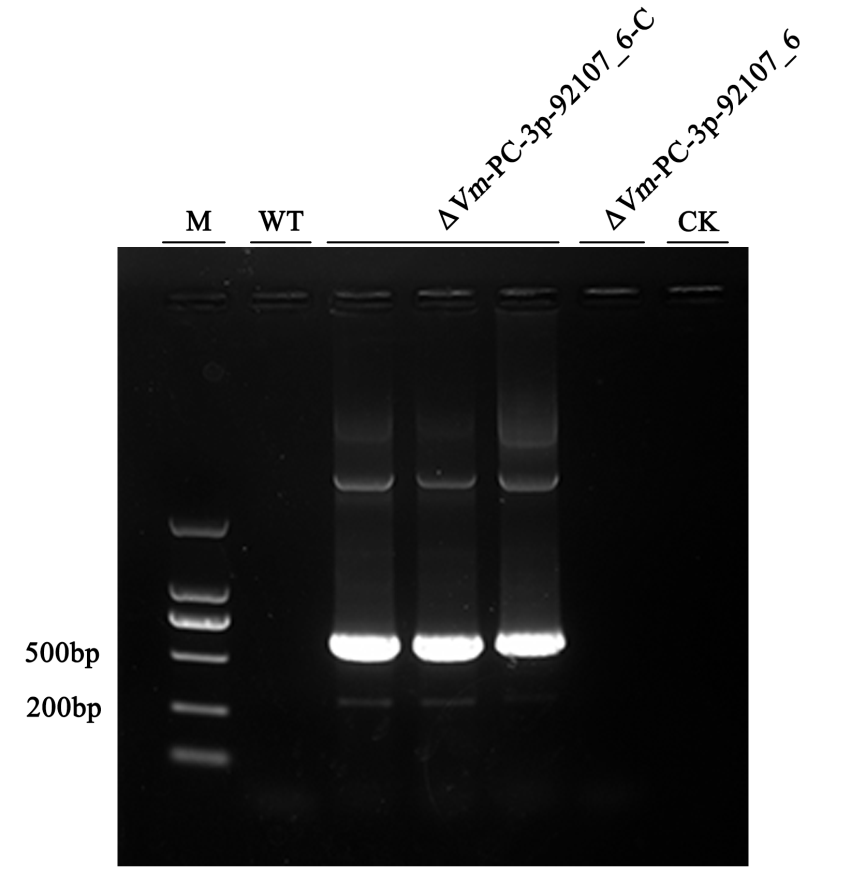


**Supplementary Figure 4.** Construction of *Vm*-PC-3p-92107_6 complement transformants (Δ*Vm*-PC-3p-92107_6-C).


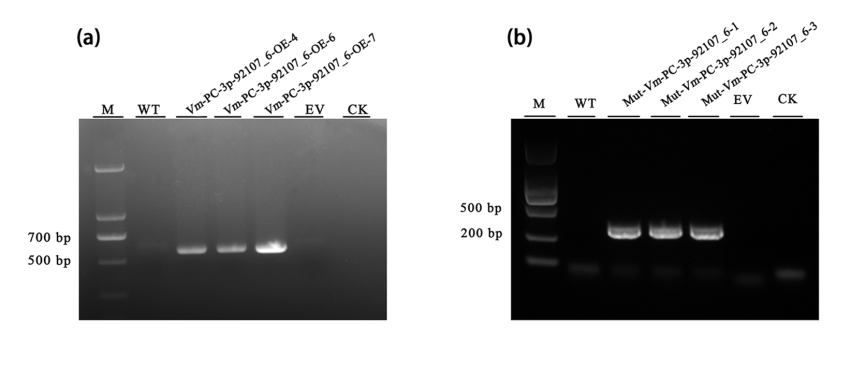


**Supplementary Figure 5.** Construction of *Vm*-PC-3p-92107_6-OE and Mut-*Vm*-PC-3p-92107_6. (a) PCR analysis of over-expression mutants by using two primers (pDL2-mexp-JC-F and *Vm*-PC-3p-92107_6-OE-R). (b) PCR analysis of mutated *Vm*-PC-3p-92107_6 by using two primers (pDL2-mexp-JC-F and Mut-PC-3P-92107_6-OE-R). Genomic DNA of the wild-type (WT), EV and sterile double-distilled H_2_O, were used as control, respectively.


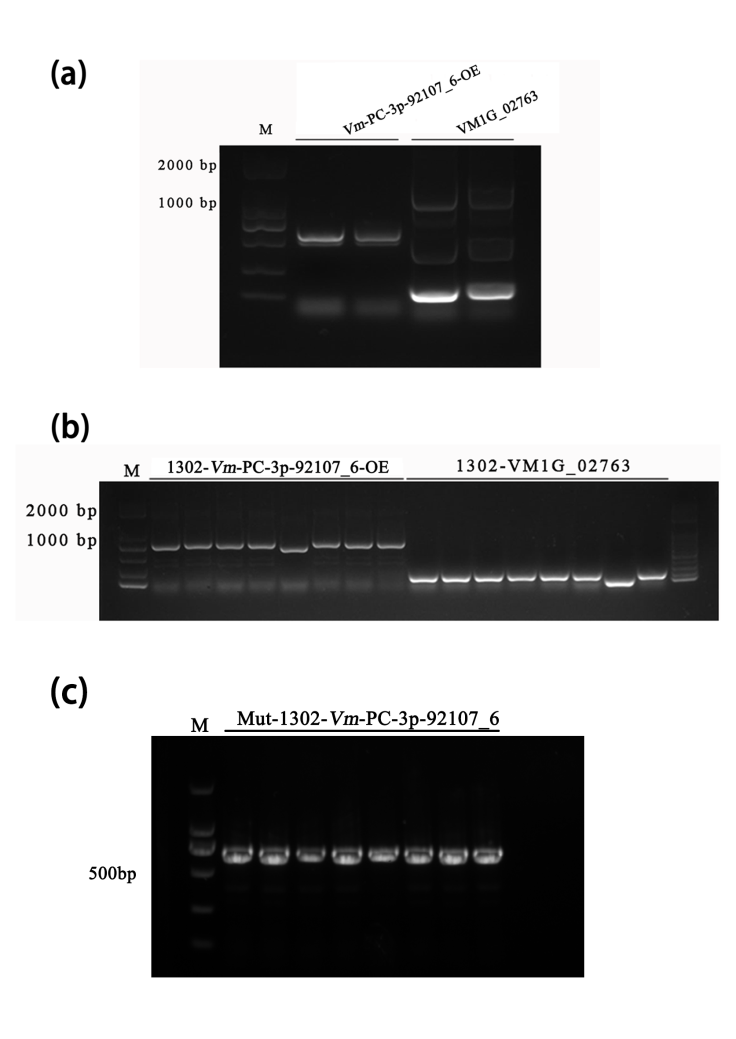


**Supplementary Figure 6.** Construction of co-expression of *Vm*-PC-3p-92107_6 and VM1G_02763 (*Vm*-*VPS10*). (a) PCR amplification of *Vm*-PC-3p-92107_6-OE2 and VM1G_02763 gene. (b) Detection recombinant co-transformation vectors.


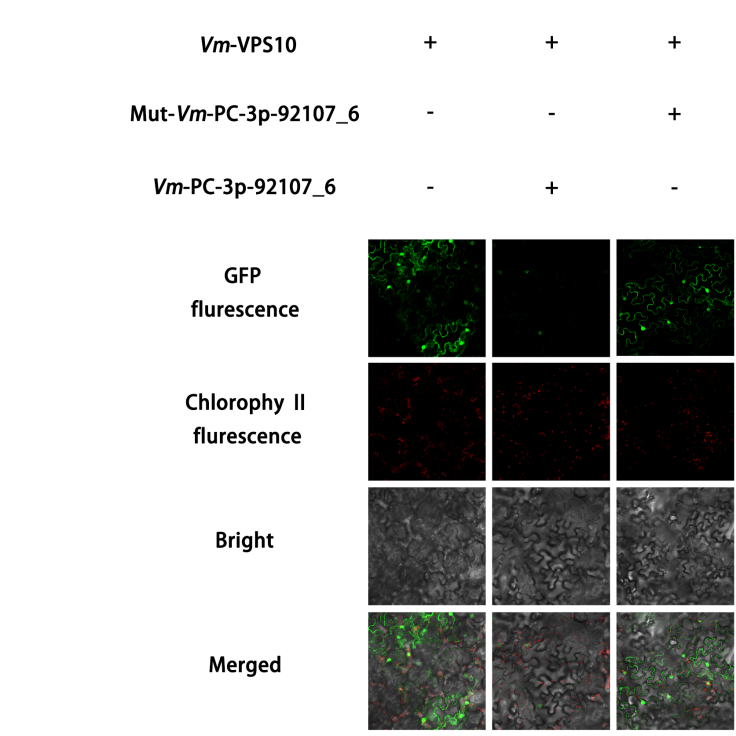


**Supplementary Figure 7.** Confocal imaging of *Vm*-*VPS10* expression vectors and *Vm*-PC-3p-92107_6 that Mut*-Vm*-PC-3p-92107_6 was used as the control. It was performed 48 hours after *Agrobacterium* infiltration.


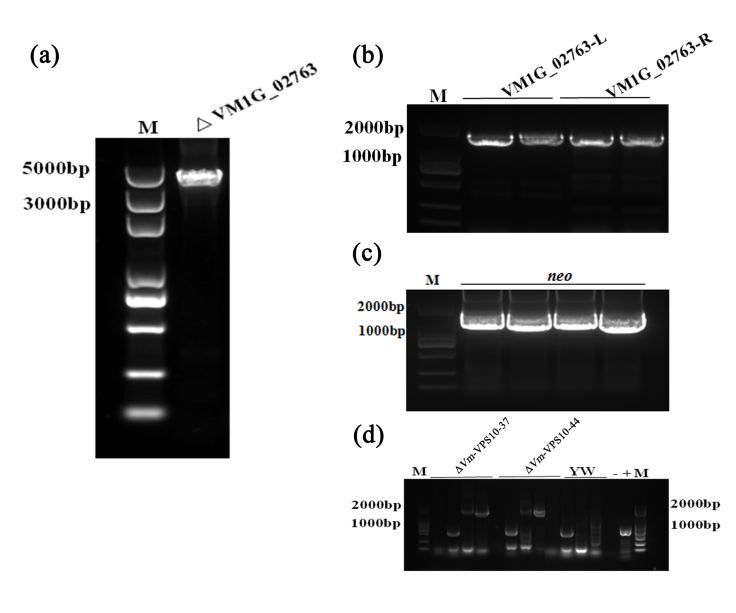


**Supplementary Figure 8.** Construction of *Vm*-*VPS10* deleted mutants. (a) Construction of deleted cassette. (b) Detection of the upstream and downstream fragment of the *VPS10*. (c) PCR amplification of Neo. (d) PCR analysis of deleted mutants by using the four types of primers (*Vm*-*VPS10*-5F/6R, *Vm*-*VPS10*-G850F/G852R, *Vm*-*VPS10*-7F/G855R, G856F/*Vm*-*VPS10*-8R). (First lane) Gene specific primers 5F/6R designed at the inner part of the target gene were used to verify the deletion of the target gene. (Second lanes) Primer pair G852-F/G850-R designed at the inner core of NEO was used to verify the insertion of NEO. Two pairs of combined primers gene specific 7F/G855-R (Third lane) and G856-F/gene specific 8R (Fourth lane) were used to ascertain the targeted homologous recombination upstream and downstream. YM: Random insertion of mutants. Genomic DNA of WT and sterile double-distilled H_2_O were used as positive and negative control, respectively.
