# Supplementary figures and images for: A Novel DCL2-Dependent Micro-Like RNA Vm-PC-3p-92107_6 Affects Pathogenicity by Regulating the Expression of Vm-VPS10 in Valsa mali
Source: Front Microbiol. 2021 Oct 1;12:721399. doi: 10.3389/fmicb.2021.721399 (PMC8575173; doi:10.3389/fmicb.2021.721399)

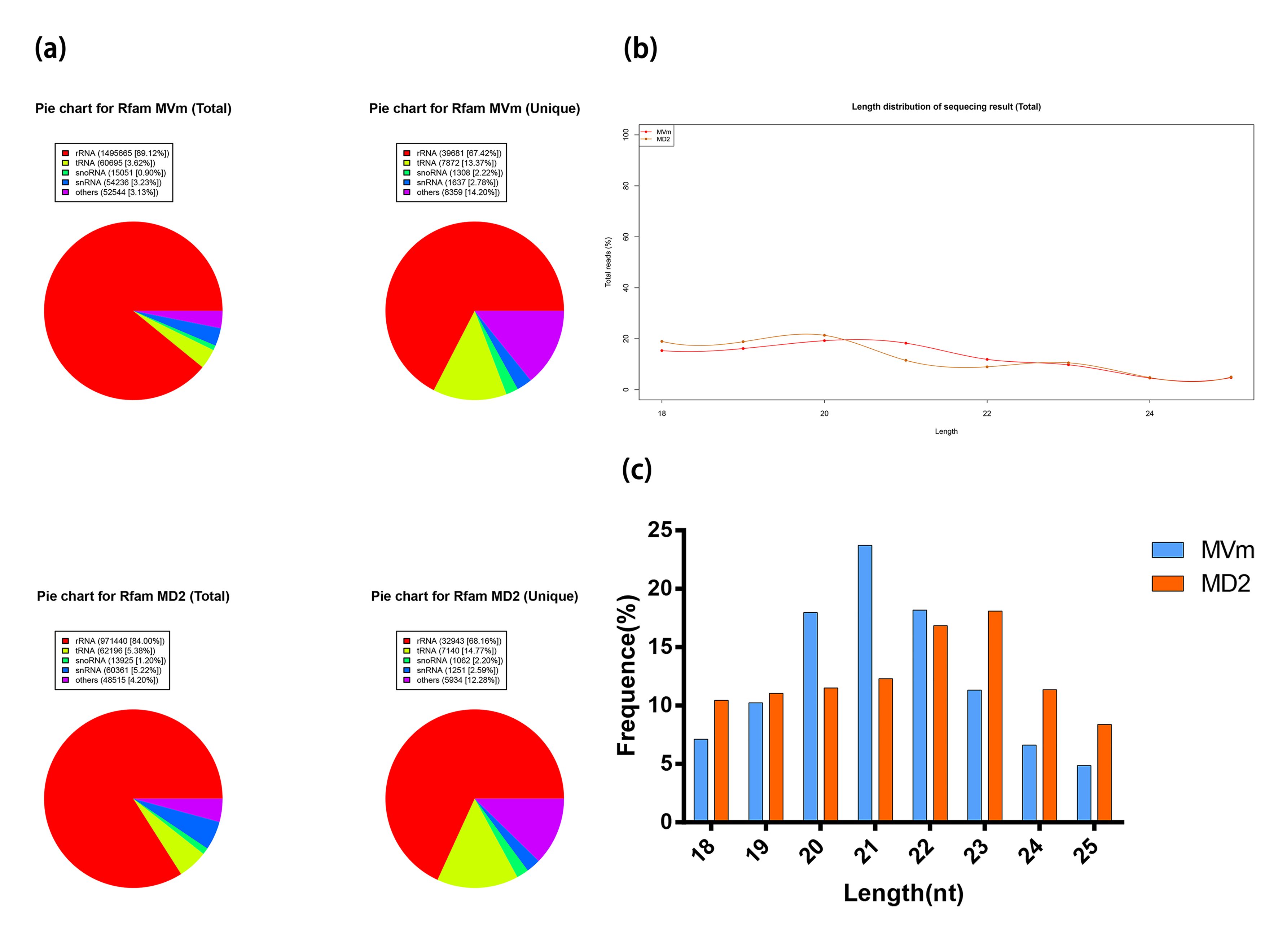

Supplement: Supplementary file 1 [file Presentation_1.ZIP › Presentation_1.ZIP/Supplementary-Figure.S1.tif]

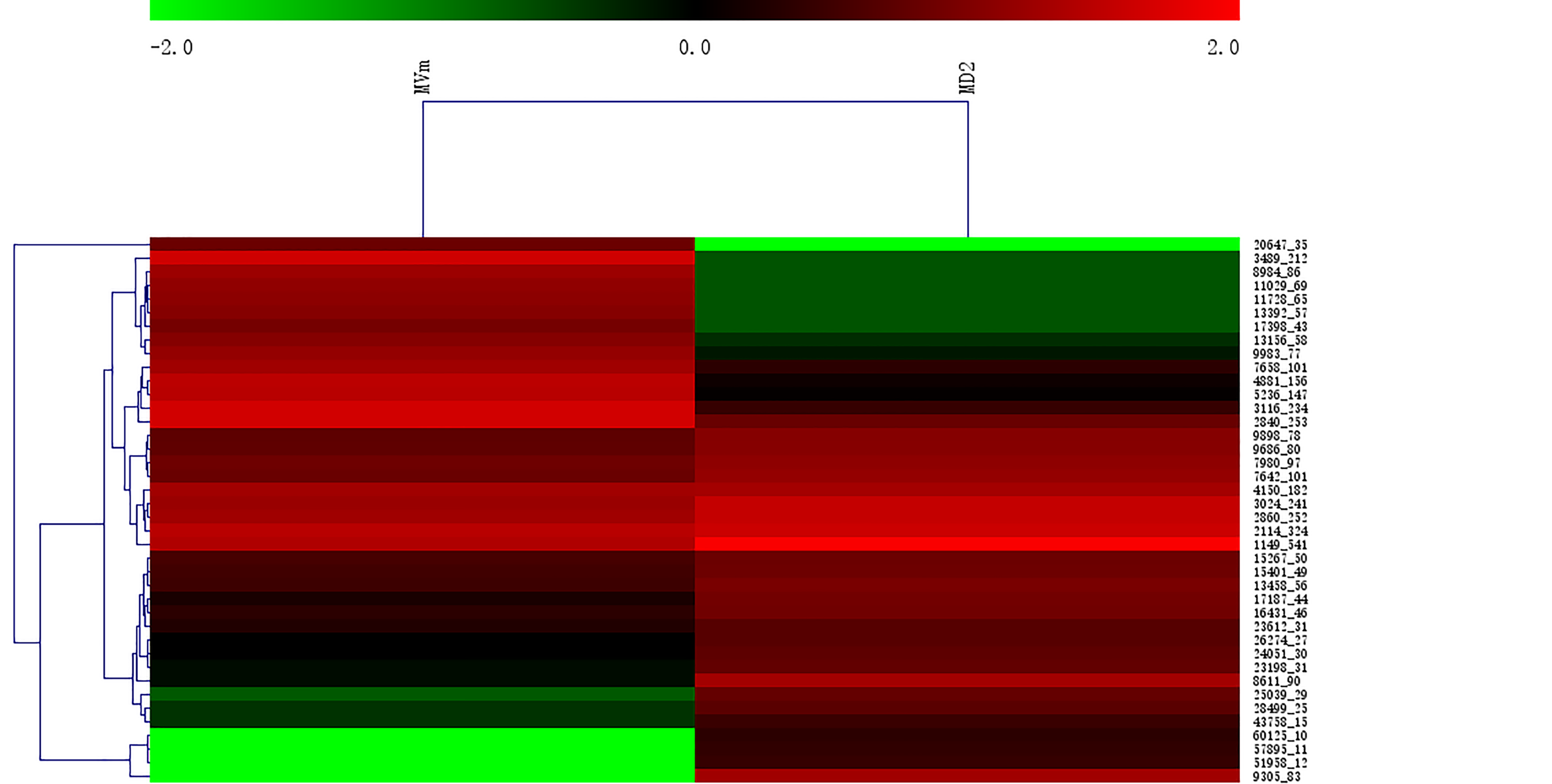

Supplement: Supplementary file 1 [file Presentation_1.ZIP › Presentation_1.ZIP/Supplementary-Figure.S2.tif]

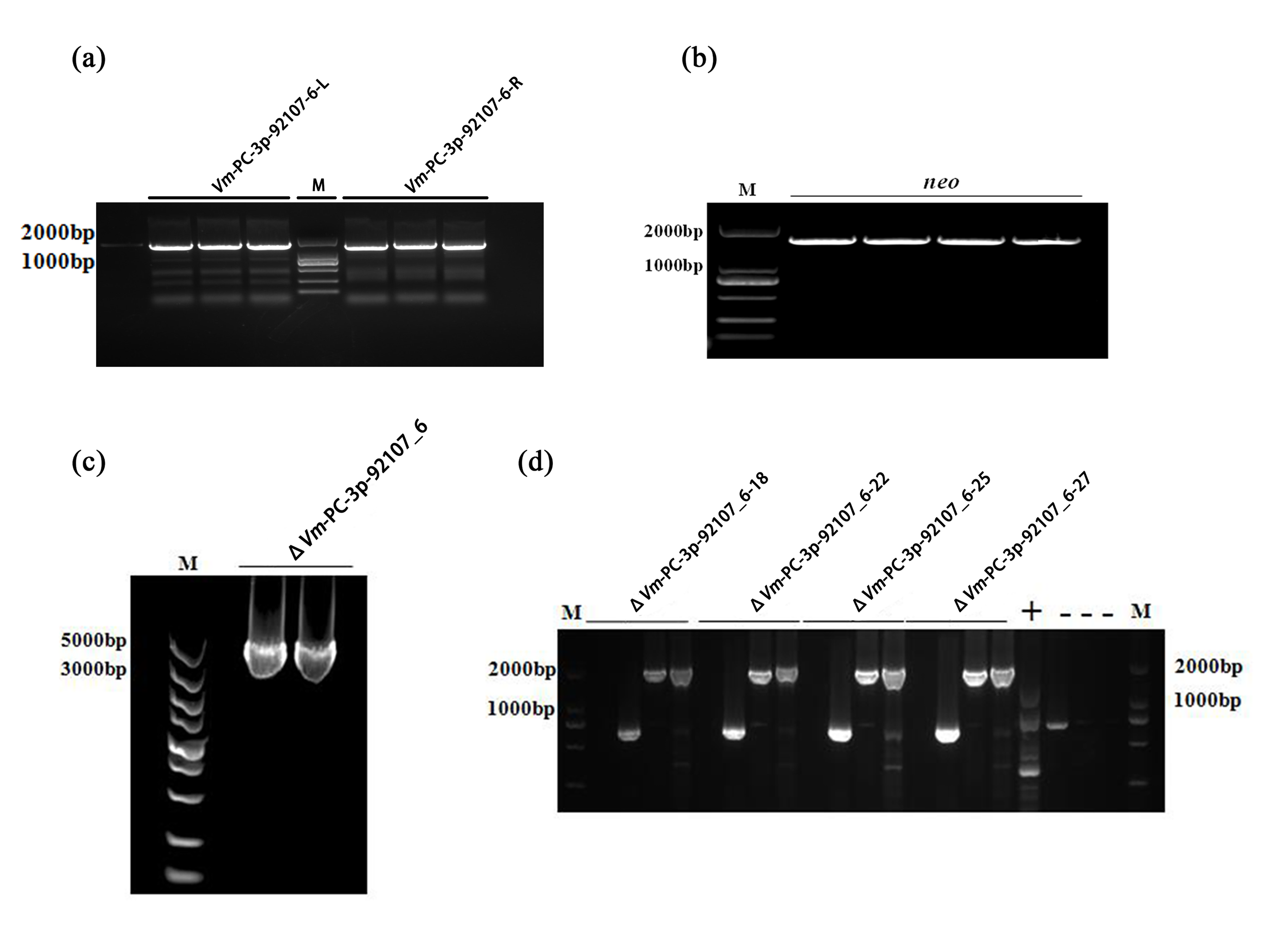

Supplement: Supplementary file 1 [file Presentation_1.ZIP › Presentation_1.ZIP/Supplementary-Figure.S3.tif]

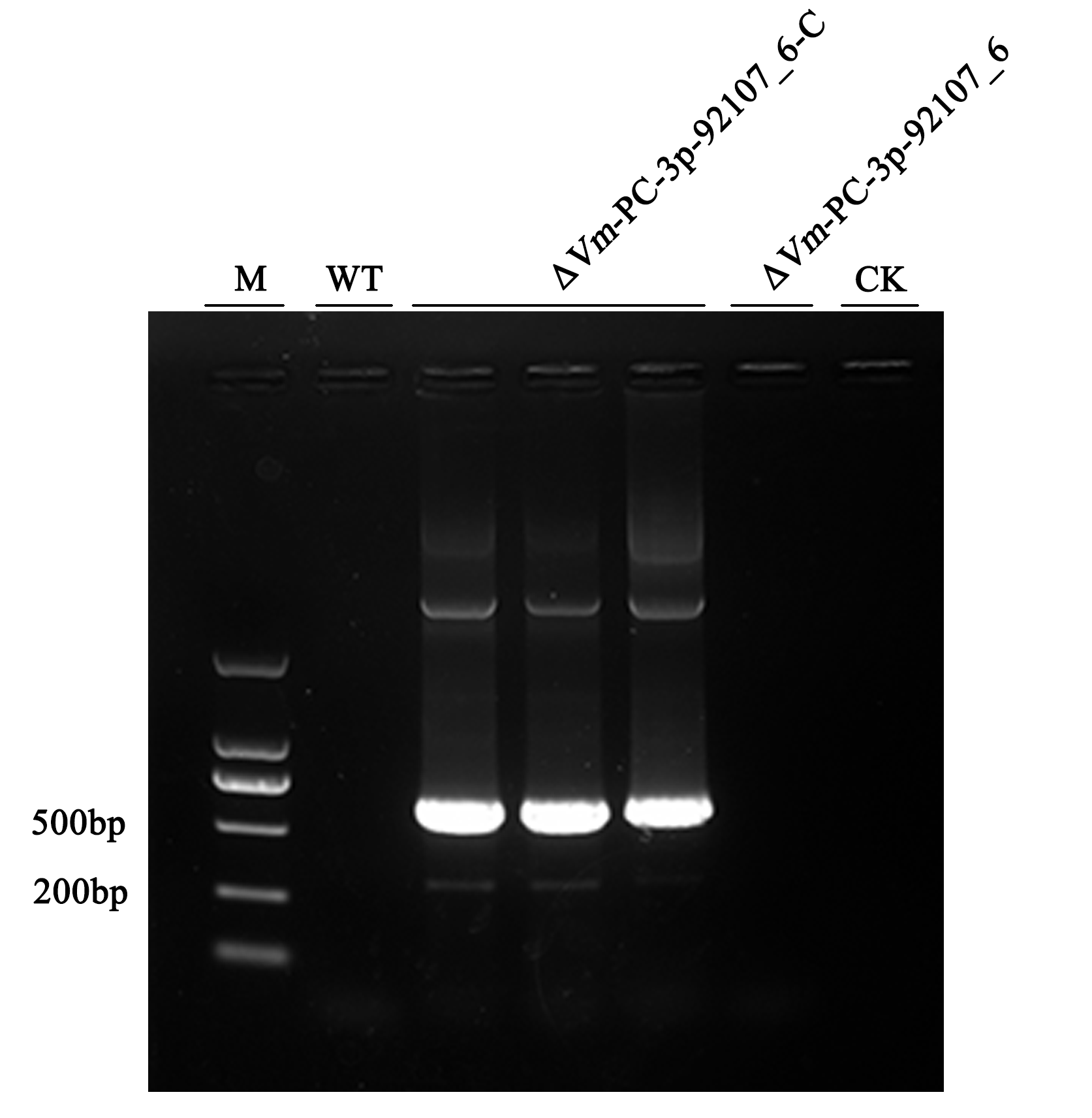

Supplement: Supplementary file 1 [file Presentation_1.ZIP › Presentation_1.ZIP/Supplementary-Figure.S4.tif]

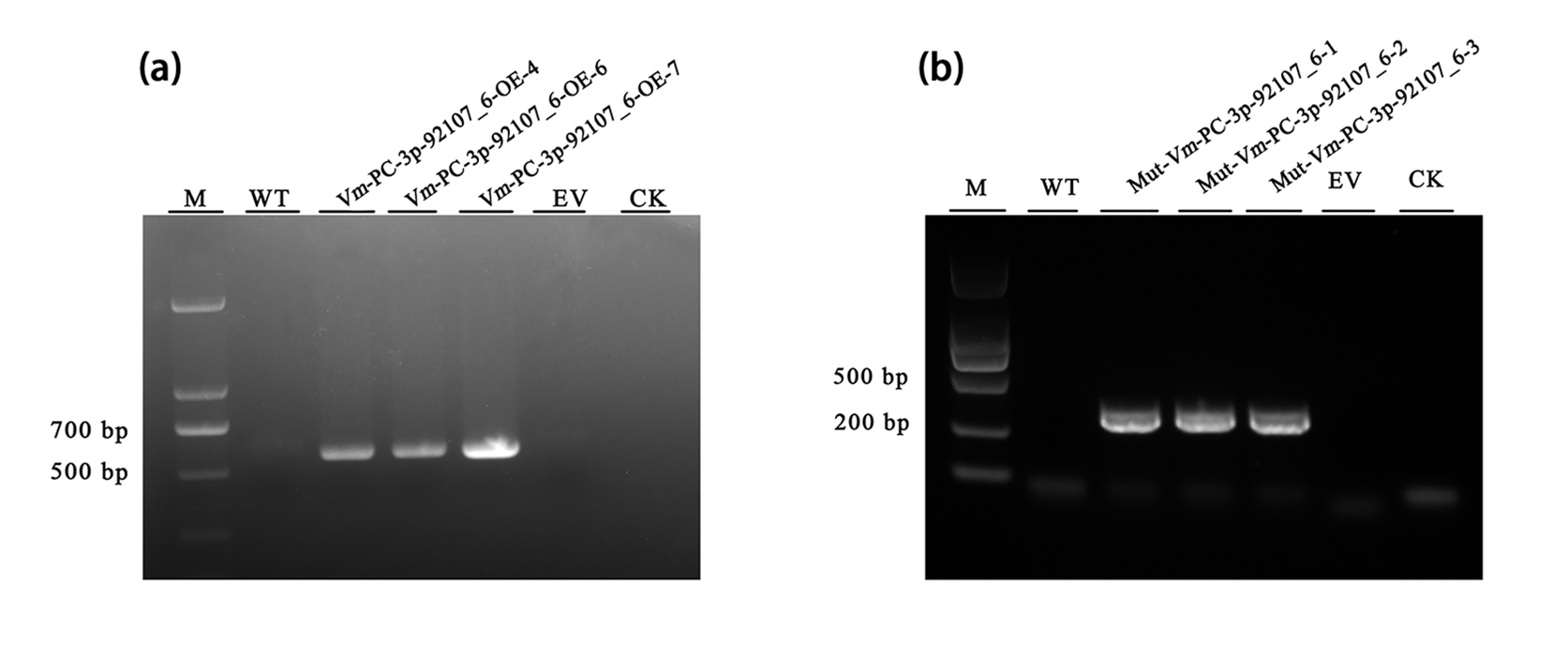

Supplement: Supplementary file 1 [file Presentation_1.ZIP › Presentation_1.ZIP/Supplementary-Figure.S5.tif]

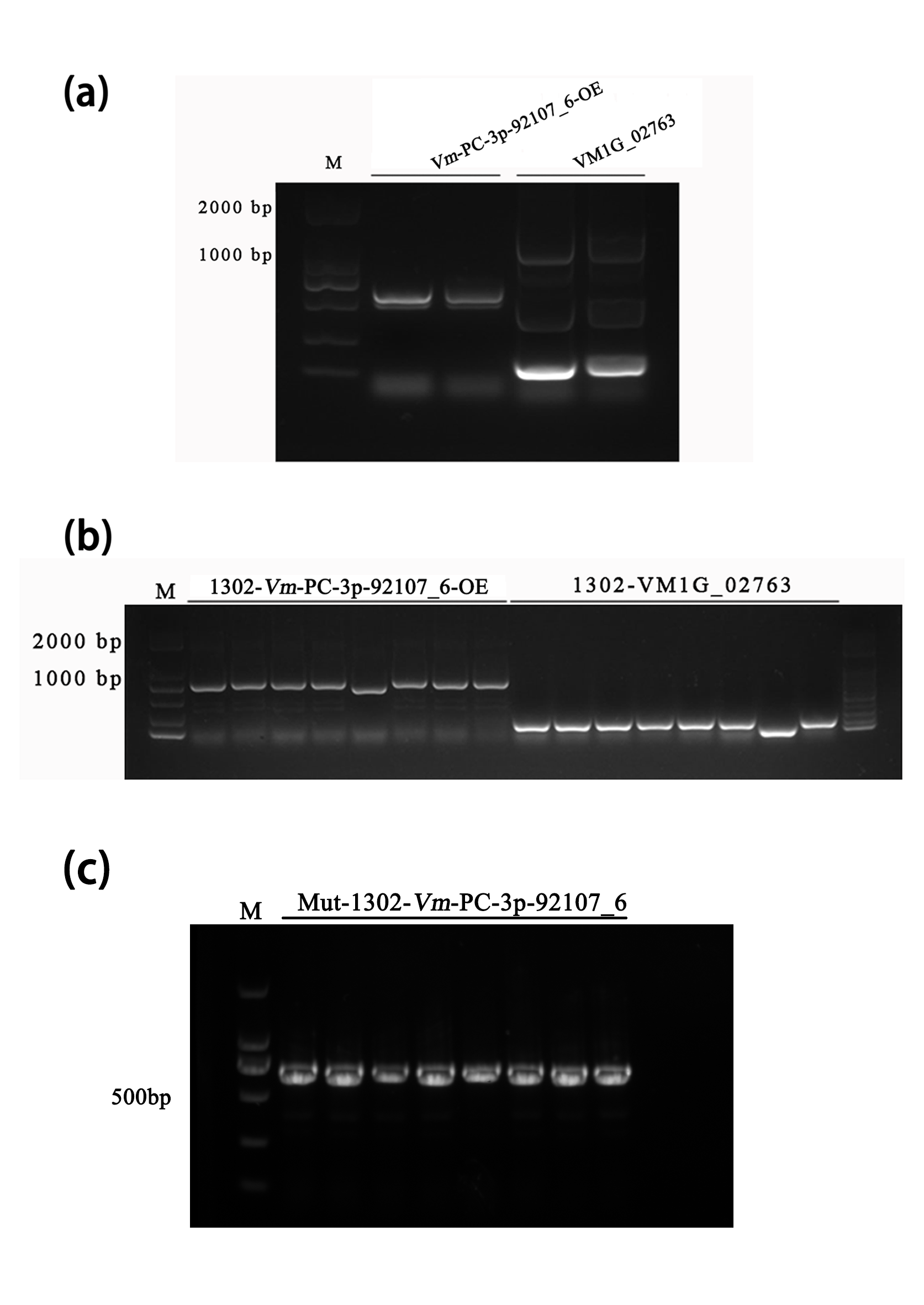

Supplement: Supplementary file 1 [file Presentation_1.ZIP › Presentation_1.ZIP/Supplementary-Figure.S6.tif]

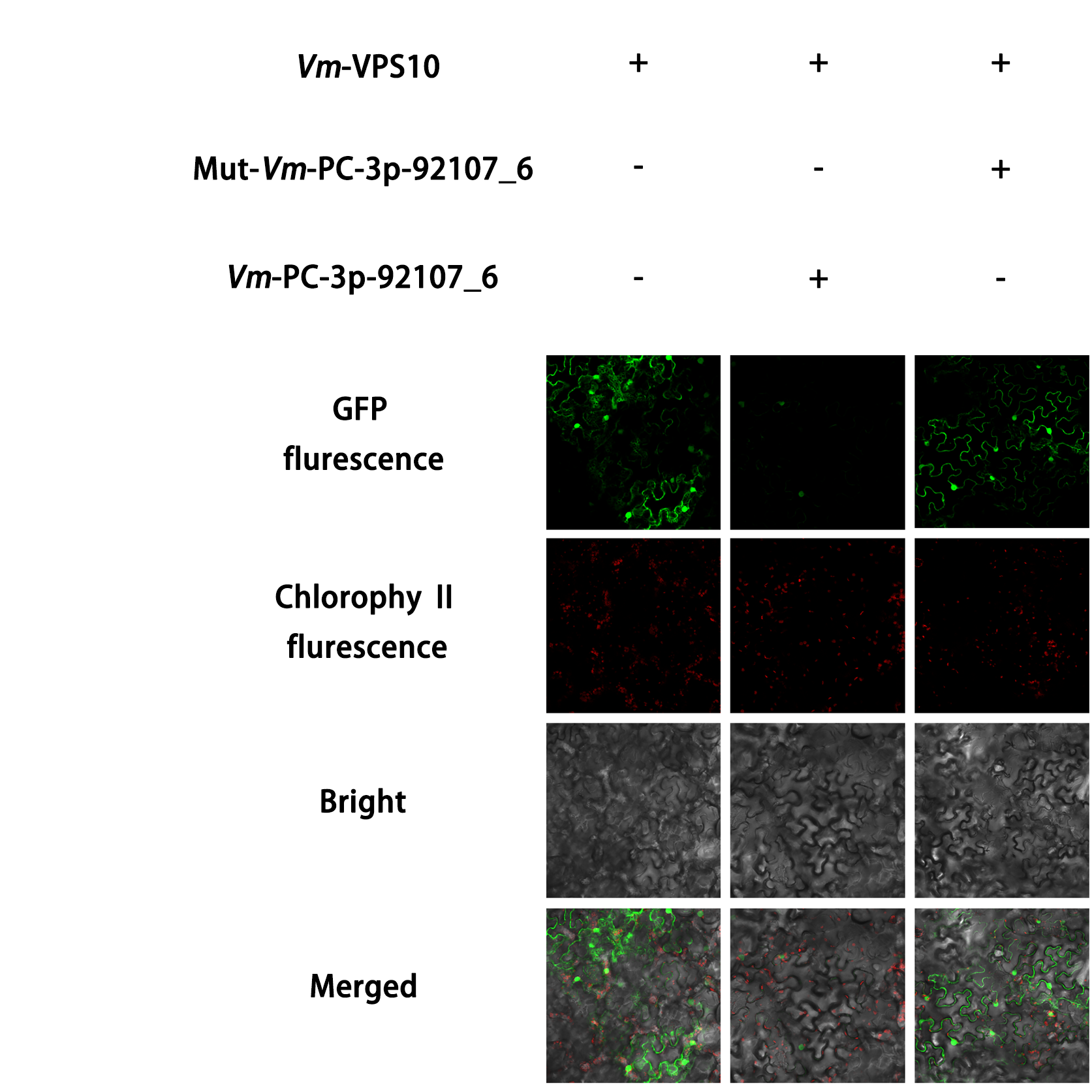

Supplement: Supplementary file 1 [file Presentation_1.ZIP › Presentation_1.ZIP/Supplementary-Figure.S7.tif]

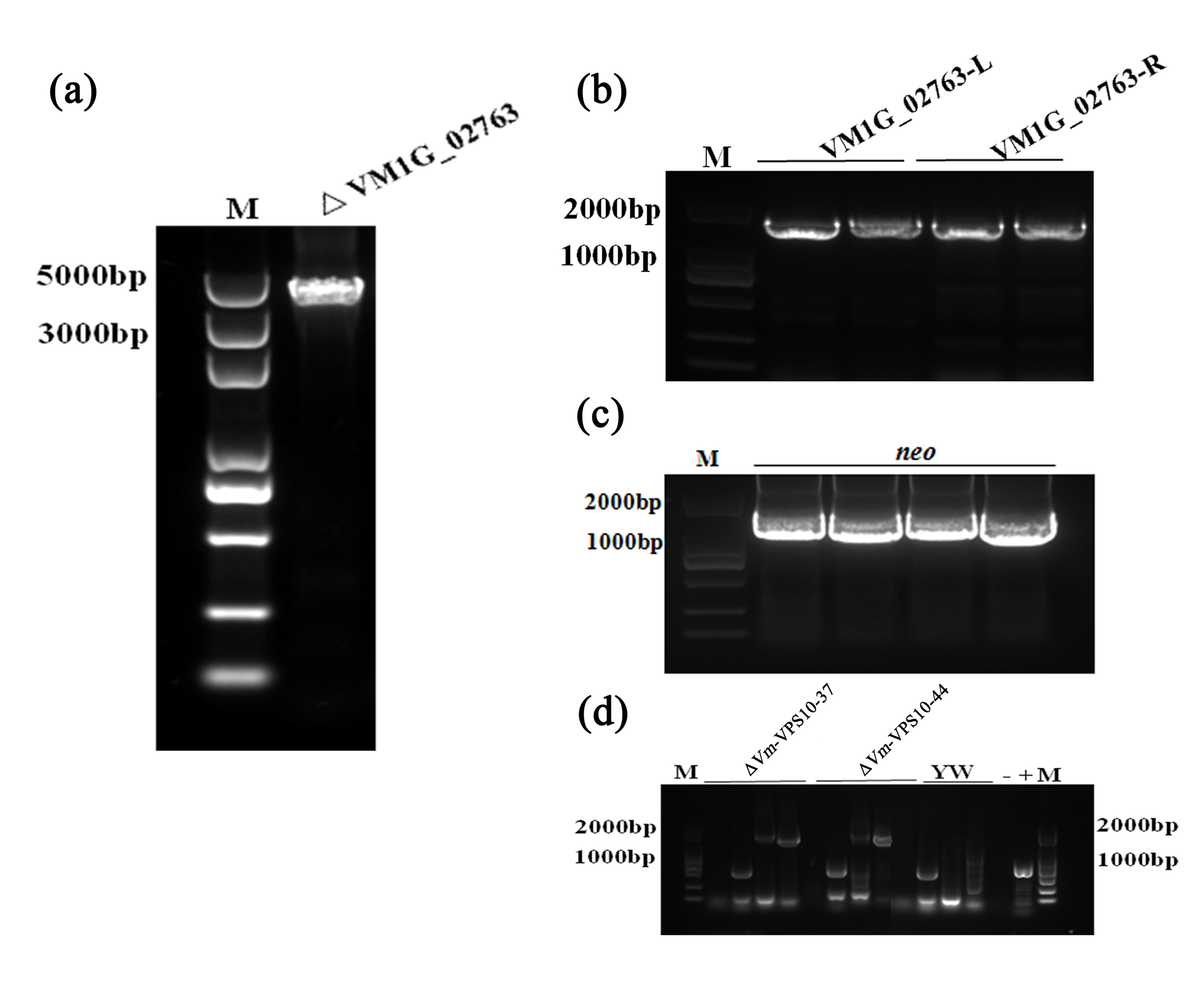

Supplement: Supplementary file 1 [file Presentation_1.ZIP › Presentation_1.ZIP/Supplementary-Figure.S8.tif]
